# Pathological and Surgical Remarks on the Diseases of the Joints

**Published:** 1834-07-01

**Authors:** 


### Medico-Chirurgical Review,

### No. XLI.

**[No. 1 of a Decennial Series.]**

APRIL 1, TO JULY 1, 1834.

### Pathological and Surgical Observations on the Diseases of the Joints. By *B. C. Brodie,* V.P.R.S. Serjeant-Surgeon to the King, and Surgeon to St. George's Hospital. Third Edition, with Alterations and Additions. Octavo, pp. 344. London, 1834.

The first edition of this work appeared, we believe, in the year 1818—the second in the year 1822—and the third is now brought before the public. Mr. Brodie informs us, in the preface to this edition, that it is not a mere republication of those which have preceded it. He observes that there are not more than two chapters which appear exactly in their former shape, and some of them are so altered that they bear but little resemblance to their originals.

We are led to offer a complete analysis of this, although an established work, on several accounts; because it is one of the most striking examples in medical literature of inductive reasoning—because the profession do not seem to be as well acquainted as they should be with its doctrines and its facts—and because we have Mr. Brodie's word for the novelty or re-arrangement of considerable portions of it.

We have said that the work is a striking specimen of inductive reasoning. We have heard this advanced as an objection. It has been urged that it displays no ornaments of diction—no grace of fancy; that all is straight and level, and uninteresting as a road in France. It is true that the charm which breathes through the writings of Pott, and the fire which blazes in the page of John Bell, do not wait on the cautious and exact demonstration of Mr. Brodie. But something more permanently useful is there—the steady pursuit and the sure acquisition of philosophical truth. Those who quarrel with the works of Mr. Brodie, turn with disgust from demonstrative reasoning; consider laborious and patient observation as the drudgery of a sordid mind, rigid deduction as the fetter of a mechanical one. They wear in their soul, if not upon their lips, the sneer of Gibbon at "the patient and sure-footed mule of the Alps." We do not purpose to eulogize nor to defend inductive reasoning, for it needs neither eulogy nor defence. We cannot refrain from deploring the absence of it in the great majority of medical works. The journalist has too frequent occasions of observing how loose and unconnected their texture is, how discreditable to the education and the capacity of the author, how derogatory to the philosophical character of the profession. It would be painful and invidious to adduce examples from the writings of those who are not of the οἱ πολλοι, but who have gained the honours and the dignities among us. This reflection leads us to single out the work of Mr. Brodie, and, by laying it fully before our readers, to inculcate its principles, evince our admiration of its execution, and hold up both to the surgical and medical world as a valuable model.

It may seem surprising that only two editions of a work so important should have been consumed in the period of sixteen years. The cause of this will be chiefly found in the prevalent unwillingness of the profession to *study* works of so grave a character. But we trust that the more complete education, medical and general, that our youth receive, with the wide diffusion of useful knowledge and cultivation of the judgment and the reason, will improve the taste of those who write and of those who read. We hope the profession will not allow twelve years to elapse before they require another edition of Mr. Brodie's treatise. It demands a moderate acquaintance only with surgical authors and with surgical practitioners, to discover that they are either unconscious or careless of the great improvements in pathology and treatment which the treatise in question contains. In recent books we often find the same vague ideas of spina ventosa, and white swelling, and scrofulous joints, which clouded the surgical horizon previous to the publication of Mr. Brodie's researches. In practice we constantly observe a lamentable ignorance in the management and diagnosis of diseases of the joints. Scarce an author appreciates to the due extent Mr. Brodie's labours—scarce a surgeon appears to be perfectly conversant with his practical improvements. Confusion obscures the pages of the one, ignorance directs the hand of the other.

On these accounts, then, we are led to offer a methodical analysis of the present edition of Mr. Brodie's work. Independently of the practical matter it contains, it is calculated to form and to improve the taste of the surgical inquirer. And we feel convinced that so many are unacquainted, or imperfectly acquainted, with what has been effected by the author, that we confer an essential boon upon the public in drawing its attention as forcibly as possible to his facts and his conclusions.*

The work consists of ten chapters, with an appendix principally dedicated to a criticism of the paper published by Mr. Key in the last volume of the Medico-chirurgical Transactions. The subjects considered in those chapters are—Inflammation of the Synovial Membranes of Joints—Ulceration of the Synovial Membrane—a Morbid Alteration of Structure of the Synovial Membrane—Ulceration of the Articular Cartilage—a Scrophulous Disease of the Joints, having its origin in the Cancellous Structure of the Bones—Caries of the Spine—Tumors and Loose Cartilages in the Cavities of the Joints—Malignant Diseases of the Joints—some other Diseases of the Joints—Inflammation of the Bursæ Mucosæ. It must be owned that this enumeration of affections displays an ample field for accuracy of diagnosis, and appropriate application of treatment. We have already stated that our notice will be strictly analytical.

* As this article was written subsequently to the completion of that upon Mr. Wickham's book, some repetitions may be noticed in the one of what the reader may have perused in the last.

In a brief introduction, Mr. Brodie alludes to the reasons which induced him to select for investigation the diseases of the joints. Perhaps the following exercised the greatest weight with himself, and will form a satisfactory apology to the profession.

"They have scarcely met with the attention which they merit from former pathologists. The terms, white swellings, scrophulous joints, &c. have been used without any well-defined meaning, and almost indiscriminately; so that the same name has been frequently applied to different diseases, and the same disease has been distinguished by different appellations. Confusion with respect to diagnosis always gives rise to a corresponding confusion with respect to the employment of remedies; and hence I was induced to hope, that, if it were possible to improve our pathological knowledge of the diseases to which I have alluded, this might lead, not indeed to the discovery of new methods of treatment, but to a more judicious and scientific application of those which are already known, and a consequent improvement of chirurgical practice." 2.

### I. On Inflammation of the Synovial Membranes of Joints.

Mr. Brodie remarks, that the adipose membrane belonging to the joints may be inflamed, and that the ligaments may be the objects of primary disease. But the malady, in either case, is too rare or too vague to form a subject of distinct consideration. The synovial membrane, like other vascular organs or parts, is frequently diseased. Mr. Brodie considers in order the morbid anatomy, the causes and symptoms, and the treatment of inflamed synovial membrane.

He observes that cases occasionally, though rarely, occur, in which a joint is swollen from a preternatural quantity of fluid collected in its cavity, independently of pain or inflammation. This passive effusion may be compared to hydrocele, and has been not improperly denominated "hydrops articuli," and "hydrarthrus." But usually the swelling is attended with pain and inflammation, and is dependent on increased secretion from the surface of the synovial membrane, occasioned by the latter. In some instances, while there is still pain and inflammation in the joint, the fluid is felt indistinctly, as if a considerable mass of soft substance lay over it. Often, when the inflammation has subsided, and the fluid is no longer to be felt, the joint remains swollen and stiff; painful, when bent or extended beyond a certain point, and liable to a return of inflammation from slight causes.

Omitting the cases whose dissections explain the preceding observations and confirm the following, we pass to their summary expression. Inflammation of the synovial membrane occasions these effects:—1st, a preternatural secretion of synovia; 2dly, effusion of coagulated lymph into the cavity of the joint; 3dly, in other cases, a thickening of the membrane; a conversion of it into a gristly substance; and an effusion of coagulated lymph, and probably of serum, into the cellular texture, by which it is connected to the external parts. In other cases, Mr. Brodie has found reason to believe that the inflammation had produced adhesions, more or less extensive, of the reflected folds of the membrane to each other. He has also observed occasionally, in dissection, such partial adhesions as might reasonably be supposed to have arisen from inflammation at some former period.

In one case related, the cartilage adhered to the bone less firmly than usual at the edge of one of the condyles of the femur. In another, the cartilage covering that portion of the articulating extremity of the femur which corresponds to the patella, presented in one spot an irregular surface, as if it had been partially absorbed, but not to a sufficient extent to expose the surface of the bone below. Mr. Brodie supposes that these were the effects of the greater disease of the synovial membrane.

" In another case, in which the patient, having recovered of inflammation of the synovial membrane, died several months afterwards of another disease, I found, on dissection, that the greater part of the cartilage of the patella, and a small portion of that covering the condyles of the femur, had disappeared, and that its place was occupied by a thin yellow membranous substance adhering to the bone, and forming a distinct cicatrix. I have known many cases in which there was extensive destruction of the cartilages of a joint by ulceration, manifestly arising from neglected inflammation of the synovial membrane. That this should happen, is no more remarkable than that ulcer of the cornea should occasionally be induced by inflammation of the *tunica conjunctiva* of the eye. At the same time, I believe it will be found, in the majority of cases of caries of the joints, that the disease has begun in the harder textures, and that the inflammation of the synovial membrane by which it is accompanied, is a secondary affection, the consequence of the formation of an abscess in the articular cavity." 13.

Inflammation of the synovial membrane occasionally terminates in suppuration, without having induced ulceration of either the soft or the hard textures of the joints. Mr. Brodie has found this after injury. It is not uncommon after accidents or operations, and constitutes one form of the purulent deposites. We have seen it after fever, and in a case of aneurism of the arch of the aorta. In one instance of articular deposite, Mr. Arnott found the cavity of the knee-joint filled with a tolerably thick pus, of an uniformly reddish colour, as if from an admixture of blood. Mr. Brodie relates a case of what appears to have been rheumatic inflammation of the joints and of the pericardium, in which a similar discoloration was remarked. The synovial membrane of the right knee was full of a dark-coloured fluid; not purulent, but having the appearance of a thick synovia, tinged with blood. The synovial membrane was every where of a red colour, as if stained by this secretion, and the cartilages of the joint had the appearance of having been stained in the same manner. There were some small extravasations of blood in the cellular membrane external to the joint. Those who are acquainted with the morbid anatomy of the heart and lungs are aware, that effusions of bloody serum in the pleura and the pericardium are by no means rare. We have frequently witnessed examples of both. M. Laennec has devoted a chapter to the " hæmorrhagic pleurisy." Probably the bloody colour of the pus, in Mr. Arnott's case—of the synovia in Mr. Brodie's—and of the serum in those to which we have adverted, are analogous in principle.

We arrive at the causes and symptoms of inflammation of the synovial membrane. This may occur as a primary affection, or be consequent to inflammation of some of the other textures of the joint. At present, Mr. Brodie treats of it as a primary affection. It very seldom attacks young children —becomes less rare about the age of puberty—and is very frequent in adults. This is an important distinctive circumstance. It may constitute a symptom or effect of rheumatism, or gout—of syphilis—of abuse of mercury. But, in these cases, the disease, for the most part, is not very severe; it occasions a preternatural secretion of synovia; but does not, in general, terminate in the effusion of coagulated lymph, or in thickening of the inflamed membrane. Sometimes it attacks several joints at the same instant, and even extends to the synovial membranes, which constitute the bursæ mucosæ and sheaths of the tendons. At other times it leaves one part to attack another, and different joints are affected in succession. In other cases, the disease is entirely local—the result of an injury, of cold, or of no very evident cause. Cold is, perhaps, the most frequent agent; and, on this account, the knee is more commonly attacked than the shoulder or the hip. Where the inflammation is thus confined to a single joint, it is more probable that it will assume a severe character, and that it may be of long duration. It is likely to leave the joint with its functions more or less impaired; and occasionally terminates in its total destruction. The complaint is most usually of a chronic kind, impairing, but not altogether destroying, the functions of the joint. If not relieved in the first instance by judicious treatment, it may continue, with occasional recoveries and relapses, for months, or even years.

The symptoms are as follow; and an accurate acquaintance with them is indispensably required.

At first the patient experiences pain in the joint, which, although affecting the whole articulation, is often referred principally to one spot. The pain usually increases for the first week or ten days, when it has attained its acmé. Sometimes even now it is inconsiderable—at others it is distressing.

When the pain has existed for one or two days, the joint may be observed to be swollen. At first this depends on a collection of fluid, which, in the superficial joints, may be felt on examination to undulate. When the inflammation has existed for some time, the fluctuation is less distinct, in consequence of the thickening of the synovial membrane. In proportion as the swelling consists of solid substance, the natural mobility of the joint is impaired.

"The form of the swelling deserves notice. It is not that of the articulating ends of the bones, and, therefore, it differs from the natural form of the joint. The swelling arises chiefly from the distended state of the synovial membrane, and hence its figure depends, in great measure, on the situation of the ligaments and tendons, which resist it in certain directions, and allow it to take place in others. Thus, when the knee is affected, the swelling is principally observable on the anterior and lower part of the thigh, under the extensor muscles, where there is only a yielding cellular structure between these muscles and the bone. It is also considerable in the spaces between the ligament of the patella and the lateral ligaments; the fluid collected in the cavity causing the fatty substance to protrude in this situation, where the resistance of the external parts is less than elsewhere. In the elbow, the swelling is principally observable in the posterior part of the arm, above the olecranon, and under the extensor muscles of the fore-arm; and in the ancle it shews itself on each side, in the space between the lateral ligaments and the tendons, which are situated on the anterior part. In like manner, in other joints, the figure of the swelling, whether it arises from fluid alone, or joined with solid substance, depends in great measure on the ligaments and tendons in the neighbourhood, and on the degree of resistance which they afford: and these circumstances, though apparently trifling, deserve our attention, as they enable us more readily to form our diagnosis." 20.

Circumstances connected with particular joints deserve to be adverted to. The disease is less frequent in the hip and shoulder than in the superficial joints; the fluctuation of fluid is not perceptible, but the existence of swelling beneath the muscles is sufficiently distinct.

"When the shoulder is affected, there is pain accompanied with a general tumefaction of the part; and, in most instances, if the hand be placed upon it, at the same time that the limb is moved, a crackling sensation is observed, which probably arises from an effusion of fluid into the cells of the neighbouring bursæ. After some time the swelling subsides, or the joint may even appear to be smaller than natural, in consequence of the muscles, especially the deltoid, having become wasted from want of exercise.

When inflammation attacks the synovial membrane of the hip, there is an evident fulness of the groin, and, in some instances, of the nates also. There is pain, which is referred, not to the knee, as in cases of ulceration of the cartilages, but to the upper and inner part of the thigh, immediately below the origin of the *adductor longus* muscles. The pain is aggravated when the patient stands erect, and allows the limb to hang, without the foot resting on the ground. It is also increased by motion, but not by pressing the articulating surfaces against each other, so that it does not prevent the weight of the body being borne by the affected limb. The pain is often very severe, yet it does not amount to that excruciating sensation which exhausts the powers and spirits of the patient in whom the cartilages of the hip are ulcerated." 21.

Mr. Brodie cannot doubt, from cases which have fallen under his observation, that inflammation of the synovial membrane of the hip occasionally terminates in dislocation of that joint, the round ligament being separated from one of its attachments by ulceration.

After inflammation of the synovial membrane has subsided, the fluid is absorbed, and, in some instances, the joint regains its natural figure and mobility; but, in other cases, stiffness and swelling remain. Sometimes the swelling retains the form it assumed whilst fluid was contained within the joint; this probably depends on the inner surface of the synovial membrane being lined with a thick layer of coagulable lymph. At other times, the swelling has nearly the natural form of the joint, when, perhaps, the synovial membrane is thickened. In whatever way enlargement may be perpetuated, the patient is very liable to a recurrence of the inflammation from exposure to cold, unusual exercise, or indeed, without any evident reason. When the synovial membrane continues thickened, it occasionally happens, that not only a certain degree of inflammation still continues to linger in the part, but that the morbid action invades the other tissues, and terminates in ulceration of the cartilages and suppuration in the joint. In such a case, the history must form the foundation of our diagnosis, and amputation is generally the only remedy.

Sometimes inflammation of the synovial membrane is more acute than that which has been drawn. The swelling takes place immediately after, or at the same instant with, the first attack of pain; there is redness of the skin: the pain is more severe; and it is so much aggravated by the motion of the parts, that the patient keeps the joint constantly in the same position, and usually in an intermediate state between that of flexion and extension. In addition to these symptoms, there is more or less of symptomatic fever of the inflammatory kind. In a few days the disease, if left to itself, assumes the chronic form; or, perhaps, under proper treatment, it subsides altogether. The boundaries of acute and chronic inflammation are too ill defined to admit of accurate description. The intermediate varieties of disease must be left to the recognition of the practical and the observant surgeon.

Inflammation of the synovial membrane is modified, as might be imagined, by the state of the patient's constitution. Where the disease is strictly local, it is generally more severe.

In syphilitic cases inflammation of the synovial membrane occurs under two conditions—in the early stage of syphilis, and usually in combination with the papular eruption, when there is little pain, inconsiderable effusion, and, after the absorption of the latter, restoration of the joint to nearly its natural condition—in an advanced stage combined with nodes, productive of much more inconvenience to the patient, more difficult to be relieved, leaving the synovial membrane thickened and the joint enlarged.

We have witnessed cases which do not quite tally with the foregoing description. Synovial inflammation has existed in combination with the papular eruption, with psoriasis, and with tubercle—it has been very acute, occasioning much pain, and recurring and disappearing under vigorous treatment. In combination with nodes, or at least with the series of cachectic symptoms that are commonly, but perhaps improperly, styled an advanced stage of syphilis, it has been unattended with acute symptoms, and neither demanded nor supported strong remedial measures.

In cases of rheumatism, continues Mr. Brodie, several joints are simultaneously or successively affected, and the synovial membranes of the bursæ mucosæ and tendinous sheaths are frequently involved. There is usually a good deal of pain and swelling, and the joints are often left stiff and enlarged afterwards. Where the inflammation is connected with gout, the pain is generally out of all proportion to the other symptoms of inflammation; and the patient feels as if the joint were in a vice, or forcibly torn open.

"There is a remarkable, yet not uncommon form of the disease, which may be considered as bearing a relation to both gout and rheumatism, but differing from them, nevertheless, in some essential circumstances. The synovial membrane becomes thickened, so as to occasion considerable enlargement of the joints, and stiffness, there being at the same time but little disposition to the effusion of fluid. In the first instance, the disease is often confined to the fingers; afterwards it extends to the knees and wrists; perhaps to nearly all the joints of the body. Throughout its whole course, the patient complains of but little pain; but he suffers, nevertheless, great inconvenience, in consequence of the gradually increasing rigidity of the joints, and the number which are affected in succession. The progress of the disease is usually very slow, and many years may elapse before it reaches what may be regarded as its most advanced stage. Sometimes after having reached a certain point, it remains stationary, or even some degree of amendment may take place; I do not, however, remember any case in which it could be said that an actual cure had been effected. The individuals who suffer in the way which has been described, are, for the most part, those belonging to the higher classes of society, taking but little exercise, and leading luxurious lives: but there are exceptions to this rule, and the disease occasionally occurs in hospital practice; in men, and even in females, of active and temperate habits." 27.

We now arrive at an important section—on the treatment of the disease.

In common acute inflammation of the synovial membrane, leeches and the ordinary antiphlogistic treatment are required. Where tension exists warm applications are most grateful, but otherwise cold evaporating lotions appear to produce a better effect.

For the chronic inflammation, quietude, local abstraction of blood, especially by cupping, with cold applications in the intervals of its employment—and blisters when the inflammation has been in a great measure subdued. Mr. Brodie prefers frequent blisters to one kept open. They should be large; if the joint is deeply seated they may be placed as near to it as possible: if superficial it is well to apply them at a little distance from it. The surgeon should remember that in this, as in other instances of inflammation of the deeper parts, blisters are improper until blood has been abstracted; that is, they are inapplicable to an early stage of inflammation. Mr. Brodie alludes to another remedy.

"When I have seen the knee joint much distended, I have, in some instances, ventured to evacuate the fluid by puncture; and the following is the result of my experience as to the effects of this operation :—

1st. In a thin person, if a few punctures be made with an instrument, a very little broader than a couching needle, by means of an exhausted cupping-glass applied over the punctures, a large quantity of fluid may be easily abstracted without the smallest danger, and with no inconsiderable relief to the patient. But while inflammation exists, the relief is not permanent, the fluid being rapidly regenerated; so that in a day or two, or perhaps in a few hours, the swelling is as large as ever. If, on the other hand, the inflammation be already subdued, the absorption of the fluid usually goes on so rapidly, that any more expeditious method of removing it is unnecessary. 2dly. If suppuration has taken place in the joint (not in consequence of ulceration, but from the surface of the synovial membrane,) a free opening made into it with a lancet will often be attended with the best effect. I have known, under such circumstances, anchylosis to become established almost immediately, and the patient to obtain a speedy cure with an anchylosed joint. The most prudent method of proceeding is to make a puncture with a needle first, and allow a small quantity of fluid to escape, so as to ascertain its nature. If it be not simply turbid serum, but actual pus, the lancet may be used afterwards." 33.

Of course the surgeon would resort to such a measure in the spirit of caution in which it is commended. It will be seen that Mr. Brodie only approves of it in cases in which suppuration has occurred.

When the inflammation is in great measure relieved, stimulating liniments may be rubbed on twice or thrice in the day. Those contained in the Pharmacopœia do not seem to Mr. Brodie sufficiently powerful. He gives a formula for one which he prefers.

℞. Olei Olivæ, ℥iss.

Acidi Sulphurici, ʒiss.

Olei Terebinthinæ, ℥ss.

Fiat linimentum.

For private patients, the proportion of sulphuric acid in the liniment should be diminished. The effect of this liniment is to excite some degree of inflammation of the skin: the cuticle becomes of a brown colour, and separates in thick broad scales. Mr. Brodie also recommends the ointment of the emetic tartar. We have found the following an improvement on the ordinary form of this ointment.

℞. Ung. hyd. fort. ℥i.

Ant. tart. ʒiss.

Iodinæ, gr. x.

M. ft. ungentum.

The common tartar-emetic ointment produces slowly a pustular eruption. The combination we have mentioned is more speedy in its operation, occasions more general inflammation of the cutis, and gives rise to an eruption more vesicular than pustular.* Stimulating plasters are considered by our author as less convenient than stimulating liniments. They seem to be adapted to an ulterior stage of the complaint, when the patient is beginning to exercise the joint. Issues and setons may be of some service in chronic cases, but they are more serviceable when the cartilage has become ulcerated.

A great deal may be effected by rest, and by those means which tend to produce it. In the early stage of the inflammation the patient's sensations warn him to abstain from motion. But exercise at a later period is productive of less immediate inconvenience, and the patient will not submit to very rigid confinement, which, indeed, would be injurious to the health. Under these circumstances the surgeon must resort to bandages or splints. On this head we may extract Mr. Brodie's sentiments.

"If the disease be far advanced, and there is danger of the cartilages being ulcerated, he will find it prudent to restrain the motions of the joint altogether, by the application of pasteboard splints, confined by a roller, or even by circular stripes of adhesive plaster on their outside. In other cases, the bandages, &c. recommended by Mr. Scott, in his ingenious work on the diseases of the joints, will be productive of the best results.† There is a bandage which is very well suited to cases of this kind, which, in one part of its circumference is composed of a stiff leather, elsewhere of an elastic material, and secured by a lace or buckles, so that it admits of being secured with any degree of tightness. If the seat of the disease be in the knee, there may be a single piece of leather adapted to the shape of the posterior part of the limb; if it be in the elbow there may be a double piece of leather, one on each side, and thus the construction of it may be varied so as to adapt it to any of the other articulations. In some instances much support may be wanted, and the leather should be stiff and unyielding: extending a considerable way above and below the joint. In others, where little support is necessary, the leather may be more pliant, and it should not extend beyond the immediate neighbourhood of the part affected. Such a bandage is worn with the greatest comfort, and it fully answers the intended purpose. As it may be removed or applied by the patient's own hands, the use of it is quite compatible with that of the stimulating liniments which I have formerly mentioned."‡ 37.

---

* Mr. Brodie has not touched on an interesting subject of inquiry—the action of different modes of cutaneous irritation on various conditions of internal morbid action.

† "A very convenient mode of applying bandages in these cases is as follows; —Let it be supposed that the disease is in the knee. Circular stripes of leather with the *emplastrum plumbi* are to be applied round the joint, and extending some way above and below it; care being taken that a space is left for the patella; on which there ought to be as little pressure as possible. Over this a calico roller (four or five yards for an adult) may be applied, and over this again a few circular stripes of linen, spread with adhesive plaster, with another calico roller over the whole. A bandage of this kind carefully adjusted may not require to be changed for six or eight weeks, and is very convenient to the patient."

‡ "Bandages of this kind are made by Shoolbred, and Co. in Jermyn Street, and by Sparkes, in Old Bond Street."

Such is the treatment, on general principles, of acute and of chronic synovial inflammation. But Mr. Brodie alluded to those forms of the disease which are dependent on, or connected with rheumatism, syphilis, and gout. In these complaints particular remedies appear to display some peculiar influence.

In rheumatic inflammation we are accustomed to employ opium, diaphoretics, colchicum, and mercury. The indications for each particular medicine it would now be out of place to enter on. With regard to colchicum and mercury, Mr. Brodie expresses the following opinions.

"I have found reason to believe that the *colchicum* is to be preferred, where several joints are affected, and where the synovial membranes, which constitute the *bursæ mucosæ* and sheaths of the tendons, participate in the disease. In such cases, the wine of the root of colchicum may be administered in doses varying from 15 to 30 minims, three times daily, or, in some instances, the acetous extract of colchicum may be given in alterative doses of 2 or 3 grains every night. On the other hand, mercury is preferable where only one or two joints are affected at a time; but where there has been a manifest translation of the disease, either from some internal organ, or from one joint to another. The form of mercury most generally useful under these circumstances, is that of calomel combined with opium, and it should be administered in such doses as to affect the gums, or to produce some other indication of its action on the general system." 29.

In the cases, apparently allied to gout, where the patient complains of an excruciating grinding pain, or feels as if the joint were torn open, the relief from colchicum is even more remarkable than in cases of rheumatism.

Where synovial inflammation is plainly connected with syphilis, mercury is beneficial; where it coexists with disease of the bone or periosteum, and forms one of the class of cachectic symptoms, sarsaparilla is required.

In cases of the peculiar chronic disease occurring in persons of a gouty habit, and invading many joints in succession, it is necessary to pay attention to the general health, to regulate the diet and amount of exercise, and to keep the bowels gently open by mild aperient medicines. It has appeared to Mr. Brodie that patients have derived benefit from the use of acetous extract of *colchicum*, exhibited at intervals of six or eight weeks, for 10 or 12 successive nights, in small or alterative doses: and still more from the long-continued use of alkalies. The carbonate of potash usually agrees with the stomach better than the pure potash. Ten or fifteen grains may be given twice daily, in the middle of the day and evening, and continued, with occasional brief intermissions, for many months.

The treatment of the stiffness which occasionally continues after the inflammation of the joint has subsided, is the only point which remains to be considered. If its mobility is only in a slight degree impaired, we may safely leave its restoration to nature. But if there be much stiffness and thickening of the soft parts, repeated blisters, or the moxa, may be serviceable. At a still later period, friction will be useful, but it ought to be employed with caution. Whenever we discover the slightest indication of return of inflammatory action, blood should be taken from the part, and the friction should not be resumed for some time. When friction can be used with safety, it should be continued for two or three hours daily, and during a considerable period of time. The *douche* is productive of much the same consequences, and applicable under much the same circumstances as the remedy last-mentioned. Whenever friction is useful the vapour-bath is useful also, in combination with shampooing and with passive motion. All these remedies require both time and patience. Where complete anchylosis has occurred no method will effect the recovery of motion in the joint.

Mr. Brodie relates three cases illustrative of the ordinary characters and progress of inflammation of the synovial membrane. These we may omit. But the case which follows displays a peculiar feature—the sudden diminution of swelling in the knee, and supervention of affection of the head.

*Case.* A young gentleman, aged about 13, was seized in July, 1817, with inflammation of the synovial membrane of one knee. Under proper treatment the inflammatory action subsided, but left a thickened state of the synovial membrane, the joint admitting only of a limited degree of motion. He was directed to employ friction with a stimulating liniment. About the middle of November the swelling became suddenly reduced and almost wholly disappeared, but on the same day he complained of acute pain in the forehead. This went off in a few hours, but for several days it returned periodically in the form of a nocturnal paroxysm, of great severity, though of only a few minutes' duration. With active treatment the pain ceased in the course of about a week, but he was then seized with somnolency, which was soon followed by strabismus, partial blindness, and almost total cessation of speech; after remaining in this state for about a week he died.

The next case is intended to illustrate a circumstance that occasionally presents itself—a sensation, on examination of the swollen joint, as of a number of small loose substances, of a soft consistence, within the cavity of the joint, and just perceptible to the touch. Mr. Brodie suspects these loose substances to be portions of coagulated lymph, which had been effused on the inner surface of the synovial membrane, and had afterwards become detached; similar to those which are sometimes formed in the cavity of an inflamed bursa mucosa. This idea, however, has not been confirmed by dissection.

In the next case, a sensation was given to the hand, on examining the knee-joint, as of some soft loose substance formed within the joint; and a crepitus was distinguished on moving the patella from one side to the other. Under the treatment resorted to, the loose substance became no longer perceptible, and the crepitus could scarcely be distinguished. Mr. Brodie supposes that this kind of crepitus, differing from that occasioned by ulceration of the cartilage and exposure of the bones, is produced by an effusion of coagulated lymph, or an altered secretion of synovia.

In the next case inflammation of the synovial membrane of the hip ended in dislocation of the head of the femur on the dorsum of the ilium.

Mr. Brodie next proceeds to an interesting form of synovial inflammation, that connected with inflammation and discharge from the urethra or the conjunctiva. From those cases it results that the affection may commence with inflammation of the urethra, or with conjunctivitis, or with synovial inflammation. This variety is well illustrated in one of the cases related by our author.

"One gentleman (at the time when these notes were taken) had as many as nine attacks of this complaint. The first took place when he was under twenty years of age, and the others at various intervals in the course of the next twenty years. In one of them the first symptom was inflammation of the urethra, attended with a discharge of pus, although, from particular circumstances, he could not believe that he had been exposed to the risk of infection. This was followed by purulent ophthalmia, and that by inflammation of the synovial membranes. In three of the attacks, a purulent ophthalmia was the first symptom; which was followed by inflammation and discharge from the urethra; and then the synovial membranes became affected; and in the other four attacks, the affection of the synovial membranes took place without any preceding inflammation either of the eye or urethra. The disease was not confined to the synovial membranes of the joints, but those of the bursæ mucosæ were inflamed also. In some of the attacks, the muscles of the abdomen were painful and tender, and subject to spasmodic contractions; and there was an occasional impediment to breathing, which seemed to arise from a similar affection of the diaphragm. The acute form of the disease, in this case, lasted from six weeks to three months, but nearly a year generally elapsed before the use of the limb was perfectly restored. He had an attack in July, 1817; and in the beginning of May, 1818, while he was still lame, he was seized with a very violent inflammation of the sclerotic coat and iris of one eye, which was subdued by very copious blood-letting, and the exhibition of mercury. He had another attack of the disorder in 1820, and in the Winter of 1822 he became affected with an inflammation of the iris and sclerotic coat of the other eye, which was also relieved by blood-letting and the use of mercury." 57.

Mr. Brodie concludes this chapter by the narration of two cases, one tending to display the good effects of colchicum, the other those of calomel and opium. In the former the patient complained of the sensation of the joint being torn open.

### II. ON ULCERATION OF THE SYNOVIAL MEMBRANE.

When an abscess has formed in a joint, an ulcerated opening takes place in the synovial membrane, through which the matter is discharged. But Mr. Brodie has only seen two cases in which the ulceration occurred as a primary affection.

*Case* 1. A young lady, æt. 9, fell and wrenched her hip. She walked out on that day, and in the evening she went to a dance, where she was seized with a rigor, and was carried home and put to bed. Next morning she was much indisposed, and complained of pain in the thigh and knee. On the following day she had pain in the hip, and was very feverish. These symptoms continued; she became delirious; and she died just a week from the time of the accident. The only lesion discovered on dissection was the collection of about half an ounce of dark coloured pus in the cavity of the hip-joint, the synovial membrane of which, reflected over the neck of the femur, was destroyed by ulceration for about the extent of a shilling.

*Case* 2. A middle aged man, who had met with a contusion of one shoulder, was admitted into St. George's Hospital. He complained of pain and tenderness in the shoulder, and a very slight swelling was perceptible. He had fever of a typhoid character. He died in a few days. Half an ounce of thin pus was discovered in the joint. The synovial membrane bore marks of general inflammation, and in one spot, where it was reflected over the neck of the os brachii, it was destroyed by ulceration for about the extent of a sixpence.

### III. On a Morbid Change of Structure of the Synovial Membrane.

This disease consists in a morbid alteration of structure, which takes place in the synovial membranes of joints, and which, as far as Mr. Brodie has seen, is peculiar to these parts. He has never known an instance in the serous membranes, nor even in the synovial membranes of the bursæ and tendinous sheaths.

Mr. Brodie relates eight cases, in order to display the pathological condition which constitutes this affection, and the manner in which the tissue is invaded by it.

It is evident that the morbid action originates in the synovial membrane, which loses its natural organisation, and becomes converted into a thick pulpy substance, of a light brown, and sometimes of a reddish-brown colour, intersected by white membranous lines. As the disease advances, it involves all the parts of which the joint is composed, producing ulceration of the cartilages, caries of the bones, wasting of the ligaments, and abscesses in different places.

We will introduce the notes of the dissection of the joint in three cases, in proof of the correctness of this summary.

*Case* 1. In a diseased knee sent to Mr. Brodie by the late Mr. Horn, the joint contained about four ounces of a pale yellow fluid, having flakes of coagulated lymph floating in it. The synovial membrane, where it formed the loose folds extending from one bone to the other, and where it was reflected over the bones themselves, the crucial ligaments, and the fatty substance of the joint, had completely lost its natural appearance. It was converted into a pulpy substance, in most parts about a quarter, but in some parts, nearly half an inch in thickness, of a light brown colour, intersected by white membranous lines, and with red spots formed by small vessels injected with their own blood. The synovial membrane on the edge of the cartilaginous surfaces had undergone a similar change of structure, but only for a small extent. The semilunar cartilages were entire, but in a great measure concealed by the pulpy substance projecting over them. The cartilages covering the bones, in a few places were in a state of incipient ulceration.

In another case the affection of the synovial membrane was more extensive, but the ulceration of the cartilage was so slight that it might not have been noticed on a superficial examination. These cases therefore shew that alteration of the structure of the synovial membrane is the primary, ulceration of the cartilage a subsequent condition.

*Case* 2. The ligaments of the diseased joint were perfectly natural. The whole synovial membrane, except where it was reflected over the cartilages, was converted into a pulpy, elastic substance, of a brown colour, intersected by white membranous lines, in some places half an inch in thickness, in others more; and in those parts where the membrane was reflected over the bones, near the border of the cartilages, it was destroyed in spots by ulceration.

The semilunar cartilages were in a natural state, but in a great measure concealed, in consequence of their being enveloped in the mass of substance formed by the diseased synovial membrane. The cartilaginous surfaces of the femur and patella were extensively, but not entirely, destroyed by ulceration; the ulceration being greatest towards the circumference. On the internal portion of the head of the tibia, the cartilage was destroyed only for a very small extent, the ulceration being entirely confined to the margin. On the external portion of the head of the tibia, the cartilage was absorbed to a greater extent. The bones possessed their natural structure and hardness. The cavity of the joint contained matter, and sinuses which opened externally communicated with it.

In another instance, besides ulceration of the cartilage, and morbid alteration of the structure of the synovial membrane, abscesses existed in the substance of the latter, and carious surfaces of bone were exposed.

These cases illustrate in a sufficient manner the pathological characters of the complaint. Mr. Brodie remarks that in every case the progress of which he has been enabled to watch, the disease has advanced slowly, and sometimes has remained in an indolent state during a long period of time, but ultimately it has always terminated in the destruction of the joint. He has never known an instance of affection of the hip or shoulder. It is usually met with in the knee. Mr. Hodgson met with an example in the ankle, and another in one of the joints of a finger. Mr. Brodie relates a case in which, on examination, the synovial membrane was lined on its interior with a straw-coloured gelatinous substance, very intimately attached to it. The membrane bore no marks of inflammation. The cartilage was partially ulcerated and the bone exposed. Mr. Brodie thinks that the effusion was not the result of inflammation, but of some other morbid action, and conjectures that it might be an early stage of the alteration of the synovial membrane.

The symptoms of the disease are thus described by Mr. Brodie.

It generally takes place in persons who are not much above the age of puberty. For the most part it can be traced to no evident cause, but occasionally it is the consequence of repeated attacks of inflammation.

"In the origin of this disease, there is a slight degree of stiffness and tumefaction, without pain, and producing only the most trifling inconvenience. These symptoms gradually increase. In the greater number of cases, the joint at last scarcely admits of the smallest motion, but in a few cases, it always retains a certain degree of mobility. The form of the swelling bears some resemblance to that in cases of inflammation of the synovial membrane, but it is less regular. The swelling is soft and elastic, and gives to the hand a sensation as if it contained fluid. If only one hand be employed in making the examination, the deception may be complete, and the most experienced surgeon may be led to suppose that there is fluid in the joint, when there is none: but if both hands be employed, one on each side, the absence of fluid is distinguished by the want of fluctuation." 85.

The patient suffers little till abscesses begin to form and the cartilage to ulcerate; even then the pain is often less severe than when the ulceration is a primary disease, and the abscesses usually heal more rapidly and discharge less. At this period the patient becomes affected with hectic, and sinks unless the limb be removed by amputation. The progress of this disease varies in different cases. In general, one or two years elapse before it reaches its most advanced stage; but sometimes the period is much longer; and occasionally it becomes indolent, so that it remains during many months without any sensible alteration.

The diagnosis is seldom difficult. The gradual progress of the enlargement and stiffness of the joint, without pain, and the soft elastic swelling without fluctuation, are sufficiently distinctive in the majority of cases. But the disease may be confounded, under two sets of circumstances, with chronic inflammation of the synovial membrane.

"1st, When the synovial membrane has undergone a morbid change of structure, it occasionally happens that a preternatural secretion of fluid takes place at the same time from its inner surface; and the joint becomes distended, not with synovia, but with a turbid serum, having flakes of coagulated lymph floating in it, which causes the tumour to present nearly the same external characters as where the synovial membrane is inflamed. But here the swelling will not yield to that treatment, under which it would be speedily reduced if it depended on simple inflammation; and attention to this circumstance, joined with an accurate previous history, will enable us to recognize the real nature of the disease.

2dly, When the synovial membrane, after inflammation has subsided, has been left in a thickened state, and coagulated lymph has been effused into the articular cavity, the tumour, in some instances, a good deal resembles the tumour which occurs in cases of this disease; so much so, that it will be very difficult to give a correct opinion, merely from observing the present appearance and condition of the joint. The surgeon must in great measure form his judgment from the account which he receives of the origin and early symptoms of the complaint; or (when an accurate statement cannot be procured) by waiting to observe its future progress." 87.

As an illustration of the symptoms, and an exposition of the diagnostic signs laid down, we will introduce the history of a case, of which we have already given the dissection.*

*Case.* A young man, æt. 24, was admitted into St. George's Hospital, with one knee swollen to nearly twice its natural size. The swelling was prominent on the anterior and lower part of the thigh. It was soft and elastic, so that at first it appeared to contain fluid; but, on particular examination, the absence of fluid was ascertained by the want of fluctuation. The leg was kept in the half-bent state, and the joint admitted of only a very limited degree of motion. He had no pain, even when attempts were made to move the limb. The skin over the diseased part was of a pale colour, with some dilated veins ramifying in it. On each side of the joint a small orifice was observed, through which the probe might be introduced into a sinus; but the sinuses appeared to be of small extent. His general health was unimpaired. He said that, two years ago, he first experienced some pain in the knee, but it was not sufficient to prevent his going about his usual occupations. Soon afterwards the joint began to swell, and the

---

* Case 3 of those intended to display the pathological condition.

enlargement gradually increased from that period. Several abscesses had formed at different times; but the greater number of them had healed.

A few observations on the treatment conclude Mr. Brodie's account of this disease. Those few observations may be readily compressed into two brief sentences—palliation of symptoms—amputation as soon as the case demands it, and the patient will consent to it.

We now proceed to an important subject—ulceration of the articular cartilage. As very many of the diseases of the joints consist in, or tend to, this condition, it is highly incumbent on the surgeon to be familiar with its symptoms, its progress, and its treatment. Specimens of gross and injurious ignorance of the nature and the characters of this disease are too frequent in public and in private practice. We shall, therefore, pursue, in a strict analytical order, the descriptions and remarks of Mr. Brodie.

### IV. ULCERATION OF THE ARTICULAR CARTILAGES.

Mr. Brodie commences, by alluding to the doctrine which has sometimes been advanced—that the cartilages not being endowed with vascularity, ulceration, when it occurs, must necessarily have been effected by the vessels of other tissues. As this opinion has lately been supported by Mr. Key, in a paper published amongst the Transactions of the Medico-Chirurgical Society, perhaps it would be well to advert to it succinctly.*

Mr. Brodie is induced to oppose this doctrine on the following accounts. 1, The phenomena of growth seem to indicate vascularity of the cartilage. —2, Analogy would lead us to entertain a similar opinion respecting the condition of the cartilage in the adult. Thus, the transparent cornea is sometimes injected with red blood, and Mr. Brodie has seen bloodvessels extend from a diseased bone into the cartilage covering it. 3, The cartilage exhibits a power of reparation, for, although exposed to constant friction, it is not observed to be worn away, like the enamel of the teeth. 4, The cartilage undergoes an alteration of structure, being sometimes converted into a number of ligamentous fibres, each of which is connected by one extremity to the bone, while the other is loose towards the cavity of the joint. 5, In some cases, the free surface of the cartilage is removed by absorption, while that immediately attached to the bone remains unaltered. But, in other cases, the ulceration begins on the side in contact with the bone, while the free one continues smooth and perfect. Under these circumstances, the space formed by the absorption of the cartilage becomes filled up by a vascular substance resembling granulations, and uniting the bone and cartilage to each other.

In whatever way ulceration of the cartilage takes place, suppuration seldom occurs so long as it is not extensive; sometimes, indeed, it is absent when the disease has proceeded so far as to cause considerable caries of the bones. But this by the way.

But Mr. Key supposes that the ulceration of the free surface of the cartilage happens only through the medium of the vessels of the synovial

* That paper was fully noticed in the last Number of this Journal. It will presently appear that Mr. Brodie has coincided with the Reviewer, and that the arguments which he employs are of the same description.

membrane. When the ulceration is at the margin of the cartilage his theory is easily accommodated to the fact. But when it is seated in the centre, he is under the necessity, as our readers are aware, of resorting to the agency of processes of inflamed synovial membrane, which, lying in contact with the surface of the cartilage, effect its absorption. Mr. Brodie pointedly observes that, if this be correct, it establishes a new fact in pathology; there being, to his knowledge, no analogous instance. Mr. Brodie relates four cases, which militate against the views of Mr. Key. Of these the first is perhaps as striking as any, and will serve as a specimen of all.

"At page 93 of this volume," says Mr. Brodie, "I have mentioned the case of a boy in whom this partial absorption of the cartilages of the knee had taken place. In some parts the cartilage had altogether disappeared; in other parts, it had been absorbed on the surface towards the cavity of the joint, while the layer next the bone remained entire; thus presenting the appearance of grooves, as if a portion of its substance had been removed by a chisel. Now, according to Mr. Key's hyphothesis, the absorption of the cartilage, in this case, ought to have been produced by villous processes of the synovial membrane projecting into the cavity of the joint, and lying in contact with the articulating surfaces. But no such villous processes existed, nor is any thing said in my manuscript notes of the synovial membrane having been even inflamed. Indeed, if it were inflamed at all, it must have been so only to a very small extent, as it is expressly stated, that there was no effusion, either of pus or synovia, into the cavity of the joint. It is to be presumed that, if the absorption of the cartilage had been effected through the agency of the synovial membrane, it would have begun, and would have made the greatest progress, at the part most exposed to contact with it, namely, at the margin; and this corresponds with Mr. Key's own observations on the subject. But, in examining the condyles of the femur taken from this patient, which are preserved in spirits in the museum of St. George's Hospital, I find that this is exactly contrary to what has really happened. Throughout nearly the whole of its circumference, for the breadth of one-third of an inch, the cartilage remains of its natural thickness, and otherwise unaltered; in the centre of the bone it has altogether disappeared, and the grooved appearance of it is observable in the intermediate space." 333.

Mr. Brodie experiences a difficulty in determining the exact mode, in which ulceration of the cartilage ensues after inflammation of the synovial membrane. But he thinks it not improbable that in many cases usually regarded as examples of simple inflammation of the synovial membrane, the inflammatory action may really have begun simultaneously in all the textures of the joint. He cites a case which seems to support this notion. Mr. Brodie also alludes to ulceration of the cartilage as a sequence of scrofulous disease of the bone—as a consequence of a change into a fibrous structure—and as a result of a chronic inflammatory affection of the bone to which it is attached.

Of course, as the cartilage is not an absolutely insulated structure, blood-vessels can only pass to it through the medium of the bone or the synovial membrane. In this sense it may be said that the cartilage cannot ulcerate except through the agency of the vessels of the other tissues. But this is a mere logomachy, or a verbal quarrel, for we might as well contend that the skin could not be ulcerated, unless by the vessels of the cellular tissue, nor the bone be altered, but through the operation of the vessels of the periosteum or medulla.

We arrive at a subject of a more immediately practical description—the causes of ulceration of the articular cartilages.

1, It may be the consequence of disease originating in the neighbouring soft parts, especially of inflammation of the synovial membrane;—2, It may depend on a morbid condition of the cartilage itself;—or, 3, On a chronic inflammation of the surface or substance of the bone with which it is connected. 4, It may be the result of a peculiar alteration in the condition of the cancellous structure of the bones, which is met with in scrofulous persons; and 5, It may follow the fibrous degeneration.

Ulceration of the cartilage, resulting from alteration of the cancellous structure of the bone, is separately treated of by Mr. Brodie. The succeeding observations principally relate to those cases in which the disease has originated either in the cartilage itself, or in the surface of bone with which it is connected. Mr. Brodie, adopting his usual method, details many cases illustrative of the pathological changes which constitute the disease. Those cases display the changes in question in the hip, the knee, and in other joints. We shall merely select sufficient for the purposes of explanation. And first of those cases which exhibit the characters and progress of disease of the hip-joint.

*Case* 1. A girl, æt. 24, was admitted into the hospital with symptoms of affection of the hip-joint, of two months' duration. In two months after admission she died. The foot was turned outwards, but no other alteration in the position of the limb is mentioned.

The synovial membrane was somewhat more vascular than usual. The cartilage covering the head of the femur had been destroyed by ulceration for more than half its extent; so as to expose the cancellous structure of the bone. The remaining portion of the cartilage covering the head of the femur was thinner than natural: but this was more observable in some parts than in others. Every where the loss of substance appeared to be on the surface towards the cavity of the joint; the layer of cartilage towards the bone being unaltered, except in one spot where it was ulcerated to a very small extent. The cartilage of the acetabulum was entirely destroyed, so that every where a carious surface of bone was exposed. There were no remains of the round ligament. The synovial membrane on one part of the neck of the femur was destroyed by ulceration; and here also a carious surface of bone was exposed. The bones themselves had their natural structure and hardness, not differing from healthy bones, except on the carious or ulcerated surfaces.

In another patient, who had laboured under the symptoms for four months, the cartilages covering the head of the femur, and lining the bottom of the acetabulum, were destroyed by ulceration for about one-half of their extent, exposing an ulcerated surface of bone. The round ligament was readily torn in consequence of ulceration having extended to it at the part where it was inserted into the acetabulum. The bones possessed their natural texture and hardness. There was no pus in the joint.

*Case* 3. This patient died ten months after the commencement of the symptoms. When admitted, six months after that commencement, the limb appeared to be an inch and a half longer than its fellow. In the progress of the case it became, or seemed to become shorter.

"On inspecting the body, the glutæi muscles were found wasted and shrunk, and in many parts their texture was destroyed by the abscesses, which communicated with the cavity of the joint by two ulcerated openings, one on the anterior, and the other on the posterior part. The abscesses formed several sinuses in the neighbourhood of the joint, and the capsular ligament was in consequence connected to, and in some measure blended with, the other soft parts. The joint contained purulent matter. The synovial membrane was darker than natural, but otherwise had the ordinary appearance. There were no remains of the round ligament. The cartilages were every where absorbed, and the exposed surfaces of bone were in a carious state. The head of the femur was reduced to about two-thirds of its original size; and the acetabulum was rendered deeper and wider, nearly in the same proportion. At the bottom of the acetabulum there was an ulcerated opening, just large enough to admit a common probe, communicating with an abscess within the pelvis. The carious surfaces of the bones had the same dark colour and fetid smell as in other cases of caries; but otherwise they did not differ from healthy bones." 103.

These cases shew the consecutive series:—Ulceration of the cartilage—exposure and caries of the bone—pus in the joint, and abscesses connected with it—absorption, by ulceration, of the head of the femur and margins or bottom of the acetabulum. In another instance the changes had proceeded farther, the head of the femur being almost dislocated on the dorsum of the ilium. The conditions were these:

In the anterior part of the joint, a quantity of organized soft substance, resembling that of adhesions, was interposed between the head of the femur and the acetabulum, and behind this was a collection of dark-coloured pus. From these two causes the head of the femur had been separated from the os innominatum, and pushed outwards, and it had afterwards been drawn upwards by the action of the muscles, so that it was lodged on the superior part of the bony margin of the acetabulum.

Mr. Brodie thus sums up the facts, and describes the progress of the disease.

"Ulceration takes place in the cartilages; generally in that of the acetabulum first, and in that of the head of the femur afterwards: sometimes it begins in both at the same time.

2. The ulceration extends to the bones, which become carious; the head of the femur is diminished in size, and the acetabulum is rendered deeper and wider.

3. Abscess forms in the joint; which after some time makes its way, by ulceration, through the synovial membrane and capsular ligament, into the thigh or nates, or even through the bottom of the acetabulum, into the pelvis. Sir Astley Cooper has shewn me two specimens, in each of which an abscess connected with a diseased hip had burst into the rectum.*

4. In consequence of the abscess, the synovial membrane and capsular ligament become inflamed and thickened. The muscles are altered in structure; sinuses are formed in various parts; and, at last, all the soft parts are blended together into one confused mass, resembling the parietes of an ordinary abscess." 107.

---

* Abscess of the hip-joint occasionally opens into the bladder and vagina.—Ed.

Mr. Brodie does not assert that the hip is not liable to other morbid affections, but he believes that, in the greater number of those cases to which the name of "diseased hip" is usually applied, ulceration of the cartilage is the primary condition.

Mr. Brodie proceeds with the detail of cases, displaying ulceration of the cartilage in other joints. We shall simply select an instance of the disease in the several articulations. And first of the knee.

*Case.* A man, æt. 26, was admitted into St. George's Hospital, with symptoms of disease of the right knee-joint. The leg was fixed, or nearly so, in the half-bent position. Subsequently to his admission, an abscess formed and burst. He also became affected with severe pain in the left knee, which, up to the period of his death, was never the seat of swelling.

On examining the body, the right leg was found bent so as to form a right angle with the thigh. The head of the tibia had been drawn towards the ham by the action of the flexor muscles, so that the condyles of the femur were unusually protuberant. The lateral ligaments were in a natural state. There were no remains of the crucial ligaments, or semi-lunar cartilages. The cartilages of the tibia, femur, and patella had been entirely absorbed. The bones were carious on their exposed surfaces, but not otherwise diseased. The synovial membrane was free from all morbid appearances, except at the points of its attachment to the bones; where, in a few places, coagulated lymph had been effused on its surface.

The left knee, externally, had its natural appearance with respect both to form and size. The leg admitted of complete flexion and extension. On dissection, the ligaments and synovial membrane were found in a perfectly healthy state; but about one-third of the cartilaginous surfaces of the tibia and femur was destroyed by ulceration, the ulceration having taken place principally, but not entirely, near the circumference. The cartilage of the patella and the semilunar cartilages were entire; but the latter, in some parts, were softer than usual. The bones were free from disease. There was no pus or other fluid in the joint.

Mr. Brodie incidentally remarks, that the condition of the left knee explains the nature of, at least, many cases of that species of white-swelling described by authors, in which there is long-continued and severe pain in the joint prior to the appearance of any tumor.

In the next case, the disease was situated in the joints of some of the bones of the tarsus.

*Case.* A female, æt. 40, was received into St. George's Hospital on account of a disease in her right foot, which had originated in a sprain twelve months previously. An abscess had formed, and a sinus led to exposed bone. The leg was amputated seventeen months after the commencement of the disease.

On examining the amputated foot, the cartilages of the joint formed by the astragalus and os naviculare were found destroyed by ulceration, and a portion of the astragalus was dead, and undergoing the process of exfoliation. The cartilages of the joints formed by the cuneiform bones with each other, with the os naviculare, and with the metatarsal bones, were in like manner destroyed, and the exposed surfaces of bone were carious. The abscess communicated with the carious joints. The ligaments and synovial membrane were in a natural state, except in a few spots, where they were destroyed by the abscess. The bones possessed their natural texture and hardness. The cellular membrane of the foot contained coagulated lymph and serum.

Mr. Brodie relates a case of an interesting character—one in which the ulceration of the cartilages appears to have been the sequence of rheumatic inflammation of the bone and periosteum. We had the opportunity of witnessing the case in question; but we think it would be better to introduce it further on, in connexion with others illustrative of the symptoms of ulceration of the cartilage. We, therefore, proceed to the consideration of those symptoms, having shewn the anatomical features of the disease, and established its occurrence as a primary and an essential one. In the latter stages, whatever joint may be affected, the destruction is usually not limited to the cartilage, but involves the bones and contiguous soft parts.

*Symptoms of Ulceration of the Cartilages.* The disease may occur at any period of life; but is seen most frequently in those between the period of puberty and the age of thirty or thirty-five. In general, only one joint is affected—occasionally two or three are attacked at once, or in succession, in the same individual. Sometimes the disease is attributed to local injury—at others, no cause can be assigned—and frequently the supposed one appears to be imaginary. This disease constitutes the great majority of those cases of caries of the hip-joint which occur in adult persons; whereas in children, the joint is principally affected with that scrofulous disease, attacking the cancellous structure of the bones, which remains to be described. As the morbid changes in the hip-joint were first detailed by Mr. Brodie, when occupied with the consideration of the pathology of the disease, so he commences his account of symptoms with those exhibited by ulceration of the cartilage in this articulation.

When the disease occurs as a consequence of inflammation of the synovial membrane, its own peculiar symptoms are preceded by those of the latter affection. When it is itself the primary disease, the only symptoms experienced for some time are pain, and a slight degree of lameness in the lower limb. The pain is at first trifling and only occasional—it becomes afterwards severe and constant. Like rheumatic pain, it has often no certain seat.

"As the disease advances, the pain becomes exceedingly severe, particularly at night, when the patient is continually roused from his sleep by painful startings of the limb. Sometimes he experiences some degree of relief from the pain in a particular position of the joint, and in no other. A patient in St. George's Hospital never obtained any rest, except when he had placed himself on the edge of the bedstead, with his feet on the ground, and resting his body on a pillow, in a position between that of lying and sitting. Another patient was seen, night and day, crouching on his knees and elbows.

As the pain increases in intensity, it is more confined in its situation. In the greater number of instances, it is referred to the hip and the knee also; and the pain in the knee is generally the most severe of the two. At other times, there is pain in the knee, and none in the hip. Sometimes there is pain referred to the inside of the thigh: or even to the foot. Wherever the pain is situated, it is aggravated by the motion of the joint; but it is aggravated in a still greater degree by whatever occasions pressure of the ulcerated cartilaginous surfaces against each other. Hence the patient is unable to support the weight of the body on the affected limb ; and if he be placed on an even surface in a horizontal position, and the hand of the surgeon be applied to the heel, so as to press the head of the femur against the concavity of the acetabulum, violent pain is the consequence ; although this be done in so careful a manner, that not the smallest degree of motion is given to the hip-joint. This circumstance is well deserving of attention; and no one should attempt to give an opinion as to the nature of a disease connected with the hip, without having made an examination in the manner which has been just described." 125.

Soon after the commencement of the complaint, the hip-joint is found to be tender whenever pressure is made on it before or behind. The absorbent glands in the groin become enlarged, and sometimes suppurate. Occasionally there is a slight degree of general tumefaction in the groin. It is curious that, in some cases, there is tenderness, and even swelling, of the parts to which pain is only sympathetically referred. Mr. Brodie has observed this in the knee several times.

When the disease has existed for some time, the nates undergo a remarkable alteration in their form. They become wasted, less prominent, and present a flattened appearance, and look wider than those of the other side. In a very few cases, this increase of width is real, in consequence of the acetabulum being filled with coagualable lymph and matter, and the head of the femur being pushed out of its natural situation. In general, however, it is apparent only. The cause of the alteration in form may, in part, be the position which the patient usually assumes when he stands erect; but the principal circumstance is, doubtless, the wasting of the fleshy bellies of the glutæi muscles.

Mr. Brodie next alludes to the alterations which are presented in the length of the affected limb—it appearing augmented in the early, and diminished in the latter stages of the complaint. The former appearance is deceptive; for, if the distance between the anterior superior spinous process of the ilium and the patella, on each side, be accurately measured with a tape, no actual difference in the length of the limbs will be observed. The apparent elongation is produced by the alteration in the plane of the pelvis, the patient resting on the sound limb and advancing the affected one, which, of course, can only be effected by the depression of the corresponding side of the pelvis, and lateral incurvation of the spine. The appearance in question will often disappear in the course of a few weeks, on confinement to the horizontal posture ; but, in growing persons, the curvature of the spine may become unalterable, and the alteration in the limb may be permanently established.

"In a few cases, where the patient is in the erect position, it may be observed that the foot which belongs to the affected limb is not inclined more forward than the other, but that the toes only are in contact with the ground, and the heel raised ; at the same time that the hip and knee are a little bent. This answers to the patient the same purpose of enabling him to throw the weight of his body on the other foot ; but it produces an inclination of the pelves in the opposite direction. The crista of the ilium is higher than natural, and there is an apparent shortening, instead of elongation, of the limb on the side of the disease." 129.

In the very advanced stage of the disease, when the head of the femur has been completely destroyed by ulceration, the muscles are not prevented from pulling it upwards. The limb is then really shortened. The foot is usually turned outwards.

In other cases the shortening of the limb is attended with all the characters of a dislocation of the head of the femur on the dorsum of the ilium. In a case which Mr. Brodie relates, this circumstance was shewn, by dissection, to depend on the following anatomical condition. The round ligament was gone, and the cartilages were nearly destroyed by ulceration. The bones were carious on their exposed surfaces, but retained their natural form and size. The acetabulum was almost completely filled with pus and coagulated lymph; the latter adhering to the carious bone, and having become highly vascular. The head of the femur was lodged on the dorsum of the ilium. The capsular ligament and synovial membrane were much dilated; and, at the superior part, their attachment to the bone was thrust upwards, so that, although the head of the femur was no longer in the acetabulum, it was still within the cavity of the joint.

The shortening of the limb in the advanced stage of the disease is usually, but not always, the precursor of abscess. There is symptomatic fever, and an aggravation of all the symptoms occurs. The abscess usually presents itself in the form of a large tumor over the vastus externus muscle; sometimes on the inside of the thigh, near the middle; and occasionally two or three abscesses appear in different parts, and burst in succession. They discharge a large quantity of thin pus, and, in the worst cases, a copious suppuration continues, till the patient sinks exhausted by hectic. An adult so rarely recovers under these circumstances, that none but the most unfavourable prognosis is justified. Children recover from this advanced stage more frequently, but seldom without complete anchylosis. If suppuration has not taken place, the natural mobility of the limb is generally regained. Mr. Brodie appends a valuable note, enumerating the affections of the hip most liable to be confounded with the preceding.

1. "Inflammation of the synovial membrane.

2. The scrofulous disease, having its origin in the bones, of which I shall speak hereafter.

3. A painful nervous affection, which occurs chiefly in young females disposed to hysteria; which will also be noticed in a subsequent chapter.

4. Affections of the sciatic nerve, of the upper part of the femur, and other diseases external to the hip, are not unfrequently mistaken for disease in the joint itself, especially by surgeons of limited experience, who are misled by the wasting of the glutæi muscles, and the flattened appearance of the nates, which may occur in any one of these cases." 133.

And next of ulceration of the cartilages of the knee-joint.

There is pain in the affected joint. At first it is slight and occasional, and relieved by a few days' rest. By degrees it becomes constant and severe, particularly at night, when it rouses the patient from his sleep. The pain is referred principally to the inside of the head of the tibia; but sometimes a slighter degree of pain extends down the whole of that bone. The pain is aggravated by motion, so that the patient keeps the limb constantly in one position, and generally half bent: and he never attempts to support the weight of the body on the foot of this side.

The symptoms of ulceration of the cartilages of the knee-joint differ from those of inflammation of the synovial membrane in these respects :—First, that in the former the pain is slight in the beginning, and gradually becomes intense, which is just the reverse of what happens in the latter; secondly, that in the former, the pain is at first unattended by evident swelling, which never appears in less than four or five weeks, and frequently not until after the lapse of several months. It must not be supposed that every case of pain in the articulation, attended with little or no swelling, is one of ulceration of the cartilages. But when the pain continues to increase, and at last becomes very severe—when it is aggravated by the motion of the joint, and by the pressure of the articulating surfaces against each other, and when, after a time, a swelling such as will now be described is established, we may safely conclude that the disease is of that nature.

The swelling which attends this disease in the knee is peculiar, and depends on a slight degree of inflammation having taken place in the cellular membrane external to the joint. It is usually trifling, and appears greater than it really is, in consequence of the wasting of the muscles of the limb. It has the form of the articulating ends of the bones, that is, the natural form of the joint. No fluctuation is perceptible, nor is there the peculiar elasticity which characterizes the alteration of structure of the synovial membrane. But a few cases occur in which this disease *is* attended with a collection of fluid in the joint, and the circumstances under which they occur are thus laid down by Mr. Brodie.

" 1st. Inflammation of the synovial membrane may terminate in ulceration of the cartilages; in which case it sometimes happens, that the flum. secreted into the cavity of the joint, in consequence of the primary disease, *is* absorbed; while, in other cases, it *is not* absorbed before the peculiar symptoms of the secondary disease have shown themselves; or,

2dly. This order may be reversed; inflammation of the synovial membrane being the secondary disease, ulceration of the cartilages having preceded it, and the effusion of synovia into the joint being the consequence of it. This I supposed to have happened in the case of John Child; which will be related hereafter.

3dly. In an advanced stage of ulceration of the cartilages, where an abscess is formed, it occasions ulceration of the soft parts, and usually makes its way to the skin; but sometimes the pus is collected in the joint, distending the synovial membrane, and causing a tumour very similar to that which would arise from it being distended with synovia. In these cases, the surgeon must form his diagnosis by attending to the previous history; by observing the degree and the kind of pain of which the patient complains; and the state of his general health; and by bearing in mind this circumstance, that blisters very seldom fail in procuring absorption of the too abundant synovia, and that they never cause the absorption of pus." 136.

Dislocation of the knee, as of the hip, occurs sometimes from ulceration of the cartilages. The head of the tibia is gradually drawn backwards by the action of the flexor muscles, and is lodged in the ham. Mr. Brodie has even known this occur where abscess has never formed, the patient ultimately recovering with a stiff joint and disfigured limb.

The symptoms attending ulceration of the cartilage, in other joints, correspond very closely with those of the disease in the hip and knee. The characteristic mark is pain in the beginning of the disease, unaccompanied with swelling, and invariably increased by pressure of the articulating surfaces of the bones against each other. The pain is chiefly referred to the actual seat of the disease. But we have seen that, in ulceration of the cartilages of the hip, the pain is referred to the knee, or even to the ankle—in disease of the knee, the pain extends down to the tibia, to the ankle, and even upwards to the thigh—so, in affection of the elbow, the pain is felt, though slightly, in the fore-arm and the wrist—and in that of the shoulder there is often a painful sensation, extending down the whole of the bone to the arm. In cases of ulceration of the cartilages of the shoulder, the joint is smaller than natural, in consequence of the wasting of the deltoid muscle. When an abscess forms in connexion with this joint, it often assumes a somewhat singular appearance, when it has first penetrated through the deltoid muscle; so that Mr. Brodie has more than once known it mistaken for encysted tumor. In the advanced stage of the disease, the joint is liable to be dislocated forwards, the dislocation being sometimes only occasional, in other instances permanent; the head of the bone then rests on the anterior margin of the glenoid cavity of the scapula, and gradually makes a new cavity for itself in this situation.

"Whatever joint is the seat of the disease, the formation of abscess is always attended with an aggravation of all the symptoms. But the degree in which the general system is disturbed, when suppuration is established, depends on various circumstances; on the age and powers of the patient; on the size of the affected joint; and on its situation. An abscess connected with a deep-seated joint occasions more extensive mischief of the soft parts, before it reaches the surface, and, therefore, is productive of more serious consequences, than one which is connected with a joint which is situated superficially." 138.

The progress of the disease varies greatly in different individuals. It is generally tedious, but occasionally it is rapid. In one patient in St. George's Hospital, four months were sufficient to effect such destruction of the head of the femur and acetabulum, as to occasion a real shortening of the limb to the extent of an inch.

This concludes the sections on the history and symptoms of this disease, and we now proceed to the treatment.

Mr. Brodie observes that the general treatment must be suited to the general condition of the patient—that when fever is present, salines are required—that tonics are indicated when he is become enfeebled—narcotics when pain is severe—that the state of the digestive organs should be carefully attended to—that a course of sarsaparilla is often advantageous, especially in those cases in which ulceration of the cartilages is connected with a chronic inflammation of the bone in the neighbourhood—that when inflammation of the synovial membrane exists as a complication, the same kind of treatment is required for it in this, as in other cases—and that in one case, in which rheumatic inflammation of the bone and periosteum appeared to give rise to ulceration of the cartilages of the knee, the patient derived the greatest advantage from the internal use of mercury. Beyond these, Mr. Brodie can lay down no general rules of medical treatment, and he deems it unreasonable to expect that any medicine can be found to exercise a specific influence over a disease, the causes of which are so various in their character.

Rest is most essential. When the lower limb is affected, the patient should be confined to the bed, or, at all events, to the sofa. In most cases, a splint or some analogous contrivance, should be employed, to maintain the limb in a state of perfect immobility. The plasters and bandages of Mr. Scott probably act on this principle.

Caustic issues have been indiscriminately recommended for the cure of diseased joints. The result of Mr. Brodie's experience is, that they are particularly applicable when the cartilages are ulcerated, and much more beneficial in this than in other diseases of the joints. Mr. Brodie prefers the issue made with caustic to that produced by the actual cautery. Setons, and blisters kept open by the savine cerate, are adapted to the same description of cases as is the caustic issue.

Local or general bloodletting is occasionally productive of advantage in the early stage, when, in consequence of exercise or other causes, inflammation of the ulcerated surfaces is present. In the early stage the warm bath is sometimes beneficial. Stimulating plasters, embrocations, and liniments are inefficacious—friction is invariably injurious. The prospect of benefit from the employment of remedies, is of course much diminished when abscess has occurred.

Having premised these general observations, Mr. Brodie proceeds to offer a few practical remarks:—First, on the treatment of this disease in the hip, and afterwards in other joints, without reference to suppuration having taken place; secondly, on the plan which should be adopted where suppuration is established, and there is a collection of pus communicating with the articular cavity.

When the cartilages of the hip are ulcerated, the patient should be rigidly confined to his bed or couch. He usually prefers lying on the side opposite to that of the disease, but this position tends to produce distortion of the pelvis and lateral curvature of the spine, besides facilitating the occurrence of dislocation of the head of the femur. A pillow, placed between the knees, is calculated to obviate, in some measure, this effect. But, if possible, the patient should be placed on one of the bedsteads invented by Mr. Earle, lying on his back, with the shoulders and thighs somewhat elevated, and the latter as nearly as possible parallel to each other; sometimes it is convenient to fix the pelvis by a bandage, passing from one side of the bedstead to the other. At a later period, when, in consequence of the extensive destruction of the articulation, the muscles begin to cause retraction of the limb, Mr. Brodie has found great advantage to arise from the constant application of a moderate extending force, operating in such a manner as to counteract the action of the muscles. For this purpose an upright piece of wood may be fixed to the foot of the bedstead, opposite the diseased limb, having a pulley at the upper part. A bandage may be placed round the thigh above the condyle, with a cord attached to it, passing over the pulley, and supporting a small weight at its other extremity. He is satisfied that this tends to moderate, if not to prevent, the shortening, and the pain which results from that morbid process.

The use of the bedstead alluded to is quite compatible with the employment of any method of counter-irritation. In children, this is best effected by blisters kept open with the savine cerate. They may be applied, of a small size, on the nates, round the great trochanter and in the groin. In the adults, the same treatment is useful in the early stage of the disease, but afterwards caustic issues are productive of more benefit. The hollow behind the great trochanter of the femur is, in many respects, the most convenient situation for the application of the caustic; but, in some cases, the application of it on the outside of the hip is attended with better effects. A slough may be made with the caustic potash in the adult, half an inch in breadth and two inches in length, behind the great trochanter. If this fails in giving relief, a second slough, of a smaller size, may be made on the anterior edge of the tensor vaginæ femoris muscle; and, in some instances, though no relief is afforded by the first issue, there is great relief from the second. Mr. Brodie is not an advocate for the maintenance of the issue by means of beans; he prefers keeping it open, by rubbing the surface occasionally with the caustic potash, or with the sulphate of copper.

"The cases in which complete relief of the symptoms immediately follows the making the issue, are not very numerous. In general, there is some degree of abatement on the caustic being applied; and, in a few weeks afterwards (provided that suppuration has not taken place), if the patient continues in a state of quietude, the pain entirely leaves him. Where the pain is exceedingly severe (as it sometimes is, so as to prevent sleep during many successive nights), it is very desirable that some method should be adopted, capable of affording more speedy relief than that which can usually be obtained from the application of the caustic. If there is reason to believe that the ulcerated surfaces are in a state of inflammation, in consequence of the joint having been too much exercised, bleeding may be had recourse to. A blister may be applied to the groin, and repeated, if necessary. Blisters applied to the knee, or to the thigh, though there is no actual disease in these parts, will often occasion considerable, or even entire, relief of the pain, which is referred to these parts in consequence of their sympathy with the hip. This is a curious circumstance; but I have known it happen in so many instances, that, however difficult it may be to explain it, I can entertain no doubt of the fact. Sometimes the pain is altogether relieved by the application of the blister; at other times, I have known it leave the knee to which the blister was applied, and attack the hip." 150.

Although a caustic issue in the groin is dangerous, from the proximity of the great bloodvessels, a seton is not equally open to objections. Mr. Brodie has tried this in many instances, and he comes to the conclusion that, where the pain is very severe, the seton in the groin is more calculated to afford immediate relief than the caustic issue; but that it is not so efficacious in checking the progress of the disease as it is in lessening the violence of the symptoms, and that the issue can be better depended on as a means of cure. It is necessary to use a curved needle, in order to make the seton in the groin; it may be passed obliquely, and include from one inch and a half to two inches of integuments.

The preceding observations are more or less applicable to the disease in other joints, besides the hip. In all, perfect quietude is indispensable. The means may vary, as one or the other articulation is affected.

"In some instances when the disease is in the knee, or ankle, or tarsal joints, nothing can be done better in the first instance than simply to lay the joint on an air-pillow, which, if not much distended with air, gives an uniform, regular, and most convenient support on every side; but, for the most part, it is better to have recourse to splints made of pasteboard, or stiff leather, neatly moulded to the figure of the limb. When the disease is in the shoulder, the fore-arm should be supported by a light leathern coat, suspended from the waist or neck, and the arm should be kept constantly bound to the side; and when it is in the ankle, great advantage will often arise from the patient wearing a common wooden leg, which will enable him to take exercise for the maintenance of his general health, without aggravating the local disease." 153.

Whatever be the means employed, they must not interfere with the use of counter-irritants. When the knee or elbow is affected, we may employ the caustic issue, or the blister kept open by means of savine cerate. Mr. B. prefers the former. In the knee, a narrow slough may be made on each side of the patella—in the elbow, on the outside and inside of the joint. For the shoulder, an issue on the anterior and another on the posterior part of the joint appear to be superior to a large blister kept open by the savine cerate. In the instance of joints surrounded by numerous tendons, as the ankle and the wrist, a blister is more prudent than an issue, the latter being liable to injure those tendons which are superficial. But Mr. Brodie has, in several cases, made an issue below the internal or external malleolus. The disease has been relieved, but the remedy has occasioned such unusual irritation and distress to the patient, that it was with difficulty he could be induced to keep it open sufficiently long.

"I have seen many cases, in which the caustic issue has, in the first instance, removed all the symptoms of the disease; and yet, after some time, notwithstanding the patient has remained in a state of perfect quietude, and there has been no evident cause of aggravation, they have returned nearly in the same form as before, and with their original severity. In some of these cases, their recurrence is to be attributed to the issue itself, which, from some cause, that the present state of our knowledge does not enable us to explain, produces an effect, apparently the opposite to that which it produced when it was first made. The issue being allowed to heal, the symptoms again subside, and perhaps the patient may find himself entirely and permanently relieved before the sore is completely cicatrized. The same thing may be observed, perhaps, more frequently, where a blister has been long kept open by means of the savine cerate: and here, if the blister be of a large size, the recurrence of the pain is usually attended with a quick pulse, and a furred tongue, and much constitutional irritation, of all which the patient is relieved, when the blistered surface is allowed to skin over. It is evident that it is of much importance, and also that it may require considerable discrimination on the part of the surgeon, to distinguish when the issue or the blister begins to be injurious, and ought therefore to be persevered in no longer.

In other instances, where the symptoms have returned under the use of the caustic issue, it has appeared to me that this was to be explained in a different manner. A very small quantity of matter has been formed by the ulcerated surfaces of the joint, but not sufficient to prevent the application of the caustic from producing in the first instance very considerable benefit. But having once begun, the suppuration has continued, until a sufficient quantity of pus has been collected to occasion distention of the joint, and the reproduction of the former symptoms, in spite of the remedy which before relieved them." 156.

Such cases are not of very rare occurrence, and should teach the surgeon to be cautious in his prognosis.

The next subject which occupies the attention of Mr. Brodie is the treatment of abscess, connected with ulceration of the cartilage. This is one of an important character, especially with reference to children, as they frequently recover, even after extensive suppuration has occurred. With adults, this fortunate termination is so rare, as to render the management of comparatively less consequence.

Mr. Brodie has not found that the method of evacuating the matter recommended by Mr. Abernethy, is attended with any peculiar advantage in cases of this description. And as the abscess depends on an ulcerated condition of the cartilage and bone, it would seem to be likely, *à priori*, as is really the fact, that the cause, still remaining, would still continue operative, and that the cavity of the abscess would not be obliterated.

Mr. Brodie has been led, in some instances, to believe, that, after the application of the caustic, the tumor formed by the abscess has diminished in size. He has not, however, seen complete absorption of the pus from the employment of this or any other means, although he has witnessed cases of collections of fluid, which might readily be considered purulent, removed by the action of absorption. In the cases in which he has been enabled to investigate the nature of the fluid, it has proved to be serum rather than pus. Mr. Brodie does not advise the early opening of the abscess. He has seen it heal more readily, whether opened spontaneously or by art, when the patient has been kept for some time in a state of quietude, and the other appropriate means of treatment have been followed.

"An abscess connected with any joint, but particularly one connected with the hip, does not form a regular cavity, but usually makes numerous and circuitous sinuses in the interstices of the muscles, tendons, and fasciæ, before it presents itself under the integuments. It is, therefore, less easy to evacuate its contents than those of an ordinary lumbar abscess; and indeed it can seldom be emptied, without handling and compressing the limb, in order to press the matter out of the sinuses, in which it lodges. But this is often attended with very ill consequences. Inflammation takes place of the cyst of the abscess, and pus is again very rapidly accumulated. Small bloodvessels give way on its inner surface, the bloody discharge of which, mixed with the newly-secreted pus, goes into putrefaction, and exceedingly irritates the general system. I have seen cases, where, after a great deal of pains having been taken to obtain the complete evacuation of the contents of the abscess, and the puncture having healed, in a few days the tumour has become as large as ever, attended with pain in the limb and a fever resembling typhus in its character, and threatening the life of the patient. A second puncture having been made, a quantity of putrid fætid pus, of a reddish-brown colour, has escaped; the confinement of which had produced all the bad symptoms, which have been immediately relieved by its evacuation.

The practice which has appeared to me to be, on the whole, the best, is the following. An opening having been made with an abscess lancet, the limb may be wrapped up in a flannel wrung out of hot water, and this may be continued as long as the matter continues to flow of itself. In general, when a certain quantity has escaped, the discharge ceases; the orifice heals, and the puncture may then be repeated some time afterwards; but where the puncture has not become closed, I have seldom found any ill consequences to arise from its remaining open." 161.

Mr. Brodie again observes, that the chance of ultimate recovery is greatly diminished in the child, and still more seriously affected in the adult, by the formation of the smallest quantity of pus. But caution in abandoning treatment, and in resuming exercise, is necessary in even the most favorable cases. For the cure to be permanent, the treatment should be pursued for some time after the patient is apparently recovered. Mr. Brodie relates a striking instance, in which a relapse was the consequence of neglect of this precaution.

The mode of recovery in severe cases, is deserving of consideration. When ulceration of the cartilages has made considerable progress, the limb is seldom preserved without anchylosis. But, if the disease has been checked in a less advanced stage, the patient may recover the natural motion of the joint, even though the cartilage has been extensively destroyed. Mr. Brodie cannot deny that cartilage is capable of regeneration, but he does not appear to feel assured that it is so. He has found the bony surfaces of a joint covered by a dense membrane, in order to supply the place of the cartilage which had been destroyed—he has also seen a compact layer of bone generated on the surface which was carious—he has noticed in the place of cartilage a thin layer of hard, semi-transparent substance, of a grey colour, and presenting an irregular granulated surface—he has observed, on the articulating surface of the bones of one hip, a crust of bony matter, of compact texture, of a white colour, and not unlike marble in appearance. Of course it is not certain that these new formations were the consequence of ulceration of the cartilages, but, from circumstances to which we need not now allude, it is more than probable that they were so.

Mr. Brodie relates many cases, illustrative of the symptoms and treatment of the disease. They display the symptoms in the knee, the ankle, the elbow, the jaw. In selecting two or three, for the purpose of exhibiting the pathological characters of the complaint, we purposely omitted all reference to symptoms. We shall now relate two cases calculated to exhibit them, and also to portray the influence of treatment. In each, the knee is the affected joint.

*Case.* A young man, æt. 18, admitted Dec. 1st, 1810. The right knee was an inch and a half in circumference larger than the other. The swelling had the form of the articulating ends of the bones. The leg was half bent, and all attempts to give it motion gave great uneasiness. The pain which he experienced was great at all times, but particularly at night, when it very much disturbed his rest. He said that, eleven months previous to his admission, he had been seized with pain in the joint, so severe as frequently to keep him awake at night—that, six weeks after the pain attacked him, the joint for the first time became swollen—that, with medical treatment and perfect rest, the pain and swelling subsided so that he was able to walk about—that in the following September, having used the joint much, the pain and swelling returned, and had continued to the time of his admission.

An abscess formed, and the limb was removed by amputation on the 18th of March. The cartilages were ulcerated—the exposed bone was carious—the joint contained pus, which had made its way by ulceration through the synovial membrane—and the semilunar cartilages were nearly, the external lateral ligament completely, destroyed.

*Case.* Mary Jenkins, æt. 21, came under the surgeons' care in St. George's Hospital in November, 1809. The knee was somewhat swollen, the swelling having the form of the articulating ends of the bones, and appearing larger than it really was, in consequence of the wasting of the limb. No fluid was perceptible in the joint, nor was there any redness of the skin. She complained of violent pain, referred chiefly to the inside of the head of the tibia, and extremely aggravated by motion. She was emaciated, and laboured under a slight degree of hectic.

In May, she had received a blow upon the knee, and soon afterwards she was seized with pain in the joint, which gradually became worse.

An issue was made with caustic on each side of the ligament of the patella. The issues were kept open by means of peas; their surfaces being also rubbed with caustic every fourth day.

At the expiration of a fortnight the pain was very much abated; she was able to give some motion to the joint without much uneasiness. The swelling had nearly disappeared.

In a short time the pain was completely relieved; however, she did not quit the hospital until the September of the following year. At this time she was free from all bad symptoms, and had recovered the perfect use of the joint.

While detailing the morbid anatomy of the disease, we alluded to the fact, that ulceration of the cartilage sometimes occurs as a consequence of acute rheumatic inflammation of the bone and periosteum. We observed that we would introduce further on a case of this description, related by Mr. Brodie. We think we may advantageously do so here, in connexion with an instance illustrative of the best mode of treating this severe, and not very rare, affection.

*Case.* Sarah Holder, æt. 22, admitted into St. George's Hospital July 26th, 1827, with a diffused swelling, extending from the upper part of the right thigh to the leg, a little below the knee. The swelling was most conspicuous in the immediate neighbourhood of the knee-joint; and from thence gradually became diminished, having no defined termination either above or below. It was somewhat elastic, the skin over it appearing glossy and tense, but not redder than natural. The patient complained of exquisite pain, especially on pressure. The pain was also aggravated by every motion of the knee; nevertheless it was principally referred, not to the joint itself, but to the thigh-bone immediately above it. In addition to these local symptoms, the pulse was frequent; the tongue furred, and rather brown; the skin hot; and the countenance anxious and expressive of much suffering. The symptoms had commenced on the day preceding that of her admission. She had been much exposed, from her habits of life (she was a prostitute), to vicissitudes of temperature, and had laboured under rheumatic pains in the elbows and shoulders for a month previous to the occurrence of this complaint.

Under the use of leeches, salines, and calomel, with opium, the swelling and pain had entirely left the upper part of the thigh, but there were still some remains of both in the immediate neighbourhood of the knee. This was on the 13th of August. On the 15th she had a relapse, after accidental exposure to cold, and painful startings of the limb soon succeeded. Calomel and opium, blisters, an issue, were tried in vain. She contracted a large ulcer on the sacrum from pressure, and on Oct. 16th she died, after a severe attack of vomiting and purging, with rigors.

On examining the body, the knee-joint was found to contain neither pus nor synovia. The cartilage of all the bones which enter into the composition of the joint were ulcerated in several places, especially that of the inner condyle of the femur. A slight extravasation of blood had taken place into the cavity of the joint, apparently from the surfaces of the bone exposed in consequence of the ulceration of the cartilages. The periosteum could be easily peeled off the surface of the femur, and the bone underneath appeared to be more vascular than is natural. The stomach was distended with an acid fluid of a green colour, similar to what had been vomited on the day preceding death. The gall-bladder was full of a very pale yellow fluid. There were no other morbid appearances.

The left shoulder, to which pain had been referred for a short time previous to death, was carefully examined, but no disease was detected in it.

We should observe that in this case the rheumatic inflammation of the bone and periosteum would appear, from the symptoms, to have been subdued by the means resorted to after the patient's admission into hospital. This circumstance would account for the trifling alterations discovered in those parts after death. In other cases of this description which occurred in the hospital—in which the symptoms were more severe and more rapidly fatal—the periosteum was found separated from the femur, nearly as high as the trochanters, by pus, and the surface of the bone was in that condition, that, had the patient long survived, extensive necrosis must necessarily have resulted. This fact which our limits prevent us from relating in a more detailed and satisfactory manner, tends to render the evidence on this subject complete.

The next case is brought forward to display the treatment which succeeds in removing this disease, when not of so severe a nature. We could add from our own note-book others.

*Case.* Sarah Hansell, æt. 46, admitted into St. George's Hospital, Aug. 22d, 1822.

She laboured under pain in the left knee, and a swelling extending up the lower part of the thigh, chiefly on the anterior part. There was no effusion of fluid into the joint. The leg was bent at an acute angle with the thigh, and the patient was unable either to extend it, or bend it further. The pain in the knee was referred chiefly to the inside of the joint; it was very severe, especially at night, when it awoke her from sleep with startings of the limb. Every attempt to press the articulating surfaces of the joint against each other was productive of acute suffering, causing the patient to scream; and she could not even bear the weight of the bed-clothes on the limb. There was much symptomatic fever, with a countenance expressive of severe suffering. The tongue was white and dry, and the pulse small and frequent.

Eleven weeks previous to her admission, she had become affected with rheumatic pains in her wrists and ankles. In the course of a few days these pains subsided, but she was now suddenly seized with most severe pains in the left knee, accompanied by much fever. After two or three days more the joint appeared to be swollen, first on the inside, then in front on each side of the ligament of the patella. The swelling attained a considerable size, but gradually diminished on the abstraction of blood by leeches and cupping. The pain, however, became progressively more severe.

She had been always subject to rheumatism. The catamenia had ceased since the beginning of the attack.

Calomel and opium so as to affect the mouth, leeches, blisters, and subsequently Dover's powder and caustic issues, were the treatment employed in the hospital. On the 8th of May, 1833, she was dismissed, the knee being anchylosed in the bent position. She still experienced slight pain in it occasionally.

Mr. Brodie observed, in the commencement of his description of ulceration of the cartilages, that it is not unfrequently combined with inflammation of the synovial membrane. It is difficult to imagine that the former could proceed to any extent, without involving the necessity for the latter. And such, indeed is the fact. Sometimes the one and sometimes the other is the primary affection. Mr. Brodie relates an instance of each; but, as an accurate discrimination of symptoms would render the diagnosis unattended with much difficulty to an observant surgeon, and as the symptoms of both affections have been amply detailed, we think we may dispense with the examples of the fact, interesting and important as they are.

The fifth chapter of the work is one of almost as much practical value as the one we have now concluded. To the latter we beg, in the most earnest manner, to direct the attention of our readers. The just discrimination of the symptoms, and the proper treatment of cases of ulceration of the cartilages, are so necessary to the surgeon in extensive practice, that we tremble to think of the consequences to patients, when their medical attendants are comparatively ignorant, as we fear they not very unfrequently are, of the items of the one and the nature of the other.

### Scrofulous Disease of the Joints.

The subject of the next chapter is a scrofulous disease of the joints, having its origin in the cancellous structure of the bones. The vulgar, and too often the professional, idea of white-swelling includes the notion, that scrofula is the fons et origo of all diseases of the articulations. As Mr. Brodie remarks, it is difficult to say, with a great degree of precision, what symptoms are, and what are not, of a scrofulous character. But when we look at the diseases of individual textures, we find that some, as inflammation of the synovial membrane and ulceration of the cartilages, occur in persons of all habits, and under circumstances of great variety, while others, as the disease on which we are at present entering, is most frequently seen in combination with constitutional peculiarities, and with other morbid affections which common consent has pronounced to be characteristic of scrofula. This, we presume, is the most philosophical view of the question, and this is the view which Mr. Brodie would seem to have taken. It is true, as he observes, that all diseases of the joints are most common in persons of a scrofulous tendency; but to say that all are, therefore, necessarily scrofulous, is certainly not consistent with the rules of reasoning, nor with the practical evidence of facts. The leading argument in favour of considering an affection of the cancellous structure of the bones as *the* scrofulous disease of the articulations may, therefore, briefly be stated to be this—that it seldom or never occurs but in persons of a strumous constitution, and that very frequently it is combined with other strumous disorders. If the opponents of this view can prove that any other disease of the joints can be placed in the same category, they may then demand with reason that *it* should be admitted to the same distinction. But as we believe that they cannot prove this, we beg to hold to the opinions of Mr. Brodie, and continue to designate the following affection as the scrofulous disease of the articulations.

In this disease, the cancellous structure of the bones is the part primarily affected; in consequence of which, ulceration takes place in the cartilages covering their articulating surfaces. The cartilages being ulcerated, the subsequent progress of the disease is, in many respects, the same as where the ulceration takes place in the first instance.

As usual, Mr. Brodie first relates some cases exhibiting the morbid changes of which the malady consists, and then adds a summary expression of their value. We will first give Mr. Brodie's summary, and then select, as illustrations, a very few of his facts.

"The morbid affection appears to have its origin in the bones, which become preternaturally vascular, and containing a less than usual quantity of earthy matter; while, at first, a transparent fluid, and afterwards a yellow cheesy substance, is deposited in their cancelli.

From the diseased bone, we see, in some instances, vessels carrying red blood extend into the cartilage. The cartilage afterwards ulcerates in spots, the ulceration beginning on that surface which is connected to the bone. The ulceration of the cartilage often proceeds very slowly. I have known a knee amputated on account of this disease, in which the cartilage was absorbed, for not more than the extent of a sixpence. Occasionally, a portion of the carious bone dies and exfoliates.

As the caries of the bones advances, inflammation takes place of the cellular membrane external to the joint. Serum, and afterwards coagulated lymph, is effused, and hence arises a puffy and elastic swelling in the early, and an œdematous swelling in the advanced stage of the disease. Abscess, having formed in the joint, makes its way by ulceration through the ligaments and synovial membrane, and afterwards bursts externally, having caused the formation of numerous and circuitous sinuses in the neighbouring soft parts.

In one of the cases which have been related, thin layers of cartilage were found lying on the ulcerated surface of bone, apparently unconnected with it. In some instances, in the advanced stage of this disease, we find nearly the whole of the cartilage forming an exfoliation, instead of being ulcerated.

This scrofulous affection attacks those bones, or portions of bones, which have a spongy texture, as the extremities of the cylindrical bones, and the bones of the carpus and tarsus; and hence the joints become affected from their contiguity to the parts which are the original seat of the disease. Sometimes, however, we may trace the effects of these morbid changes even in the shaft of a cylindrical bone; so that we see the femur or tibia converted in its middle into a thin shell of earthy matter, enclosing a medullary canal of unusual magnitude.

It has been remarked by a modern author,* that, in the last stage of this disease, the bones not only lose the preternatural vascularity which they possessed at an early period, but even become less vascular than healthy bone. I believe the observation to be correct; and this diminution of the number of vessels, and, consequently, of the supply of blood, is probably (as this author has suggested) the proximate cause of those exfoliations which sometimes occur where the disease has existed for a considerable length of time, especially in the smaller bones." 194.

We have now to present some cases confirmatory of the foregoing observations.

*Case.* A lad, æt. 18, of scrofulous appearance, was admitted into St. George's Hospital in October, 1815, on account of pain, which he referred to the inside of one foot. It was constant, but slight, and did not prevent

---

* Lloyd on Scrofula, p. 123.

his walking; there was very little tumefaction. His health was bad, and he had several small ulcerations at the edges of his eyelids. In February he was seized with a fever, of which he died in March.

On dissection, the foot, which had been the seat of the pain, was particularly examined. The bones of the tarsus, and metatarsus, were found to contain an unusually small quantity of earthy matter, so that they were preternaturally soft, and admitted of being cut in any direction with a scalpel, without turning its edge. The cut surfaces of these bones were of a deep red colour, in consequence of increased vascularity; and vessels injected with their own blood could be distinctly traced extending from the bones into the cartilages covering them, and rendering the latter, in a few spots, of a red colour. The cartilage covering the internal cuneiform bone where it forms the joint with the metatarsal bone of the great toe, was ulcerated to a small extent. The ulceration had begun on that side of the cartilage which was connected to the bone; the surface towards the joint remaining entire. The bones of the tarsus were more diseased than those of the metatarsus; and those on the inside of the tarsus were affected in a greater degree than those on the outside. The bones of the other foot were affected in the same manner, but in a much less degree. Some of the other bones were examined, and were found nearly in a natural condition.

In the case which, in Mr. Brodie's work, immediately follows the preceding, both elbows, one knee, and several other joints were affected. In the next case, the disease had proceeded farther in its destructive operation.

*Case.* A young man, of scrofulous habit, was received into the hospital on account of a complaint in his right ankle and foot. There were sinuses on the outside of the latter. In five or six weeks after his admission, the leg was amputated.

On examining the foot, the cells of the cellular membrane were found distended with serum and coagulated lymph.

All the bones had undergone a morbid change, similar to what was observed in the last case, except that they were still softer and more vascular.

The cartilages of the ankle were completely destroyed by ulceration, and the exposed surfaces of bone were in a state of caries. The cartilages of the tarsus were entire, but, in some places, of a red colour; and this was found to arise from vessels loaded with red blood, extending into them from the bone. The ligaments and synovial membranes of the tarsal joints were in a natural state, as were also those of the ankle, except where they had been destroyed by the abscesses.

We do not know that it is necessary to add more specimens of the scrofulous disease of the joints. The preceding display all its phases, from the alteration of the cancellous structure of the bone to extensive ulceration of the cartilages, caries or death of the bone, and abscesses and sinuses opening externally. Those who would wish for more facts in detail, we recommend to consult Mr. Brodie's work. We pass to the consideration of the symptoms.

The disease occurs frequently in children, but is rare after thirty years of age. The hip and shoulder are less liable to it than the other articulations, probably from their being less exposed to the influence of the external cold. Several joints are sometimes affected at the same time, or in succession, a second being attacked after the first has been cured, or removed by amputation. It is seldom observed in any but in those of a scrofulous habit; and, in many cases, is preceded, attended, or followed by some other scrofulous symptoms. Mr. Brodie has often been led to believe, that the occurrence of this disease in a joint has suspended the progress of some other, and, perhaps, more serious disease elsewhere.

There is more likelihood of its being confounded with ulceration of the cartilages, than with any other disease of the articulations. But a moderate degree of practical discrimination will serve to point out the real nature of the disease in the early stage; and, when very advanced, the general implication of the textures, and the state of constitution of the patient, render the diagnosis of comparatively no importance.

"While the disease is going on in the cancellous structure of the bones, before it has extended to the other textures, and while there is still no evident swelling, the patient experiences some degree of pain; which, however, is never so severe as to occasion serious distress, and often is so slight, and takes place so gradually, that it is scarcely noticed.

After a time (which may vary from a few weeks to several months), the parts external to the joint begin to sympathise with those within it; and serum and coagulated lymph being effused into the cellular membrane, the joint appears swollen. The swelling is puffy and elastic, and though usually more in degree than it is at the same period in those cases, in which the ulceration of the cartilages occurs as a primary disease, it is not greater in appearance, because the muscles of the limb are not equally wasted from want of exercise. I have observed that, in children, the swelling is, in the first instance, usually less diffused, and somewhat firmer to the touch, than in the adult.

If a suspicion of some disease of the joint has not existed previously, it is always awakened as soon as the swelling has taken place. Should the patient be a child, it not uncommonly happens that the swelling is the first thing which the nurse or the parents discover. This leads to a more accurate enquiry, and the child is observed to limp in walking, if the disease be in the lower limb, and to complain of pain on certain occasions.

I have said, that the swelling is puffy and elastic; and, after what has been remarked in the former chapters, it is needless to point out more particularly the difference between it and the swelling which takes place in cases of inflamed synovial membrane. The swelling increases, but not uniformly, and it is greater after the limb has been much exercised than when it has been allowed to remain for some time in a state of quietude.

As the cartilages continue to ulcerate, the pain becomes somewhat, but not materially, aggravated. It is not severe until abscess has formed, and the parts over the abscess have become distended and inflamed. The skin, under these circumstances, assumes a dark red or purple colour. The abscess is slow in its progress; when it bursts, or is opened, it discharges a thin pus, with portions of curdly substance floating in it. Afterwards the discharge becomes smaller in quantity, and thicker in consistence, and at last it nearly resembles the cheesy matter which is found in scrofulous absorbent glands.

In most instances, several abscesses take place in succession, but at various intervals; some of which heal, while others remain open, in the form of fistulous sinuses, at the bottom of which carious bone may be distinguished by means of a probe." 199.

The disease may remain thus for months, or even longer, without material disturbance of the constitution. In less fortunate cases, the patient sinks from hectic, unless the cause be removed by amputation. At other times, a curative process is set up, and anchylosis is established. But the cure is always tedious unless the complaint has been arrested very early. The chance of recovery is less where the joints of the foot and hand are attacked, than where the larger, but simpler articulations are implicated.

The leading point of distinction between the symptoms of this disease and those of ulceration of the cartilages, consists in the less degree of pain attendant on the former. The patient seldom suffers much, except when an abscess is just presenting underneath the skin, and its bursting affords him immediate relief. The exhausting pain and suffering occasioned by idiopathic ulceration of the cartilages is not witnessed in this disease, except in a few instances, and in the most advanced stage, when a portion of the ulcerated bone has died, and, having exfoliated so as to lie loose in the cavity of the joint, irritates the parts with which it is in contact, and thus becomes a source of constant torment. Independent of this symptom, the general aspect and habit of the patient, the tediousness of the disease, and the circumstance of abscesses occurring in succession, are features distinctive of the malady.

The progress of this disease in the hip very much resembles that of the disease described in the last chapter. What pain there is, is referred to the knee rather than the hip. Dislocation upwards and outwards occasionally takes place; Mr. Brodie has once seen it forwards, the head of the femur resting on the pubes. Abscesses form in the advanced stage. Yet in this, as in other joints, the lesser quantity of pain will commonly enable us to distinguish this disease from ulceration of the cartilages of the hip.

"When the disease occurs in those joints which are more superficially situated, such as the knee and ankle, we may be further assisted in our diagnosis by observing the character of the swelling by which it is accompanied, and which is somewhat peculiar, especially in children, previously to the formation of abscess. It is then limited to the immediate vicinity of the affected part and has a pretty well defined margin. When the disease is in the knee, the child usually keeps the leg a good deal bent, and the condyles of the femur are seen projecting, of a somewhat globular form, and appearing as if they were actually enlarged, although we know them to be not enlarged in reality. Altogether, however difficult it may be to describe it in words, the appearance is very characteristic; so that, judging from it alone, an experienced surgeon will, in many instances, be able at once to form a correct diagnosis." 203.

We have now to consider the treatment of this disorder.

Mr. Brodie observes that, the disease being the effect of a certain condition of the general system, general remedies must of course be necessary. On those remedies we need not take the trouble to dilate, as the profession are familiar with the subject. We need only hint, that regular habits and nutritious, but simple diet—pure air—tonics—and occasional aperients, must form the most important items in any plan of management. Of iodine Mr. Brodie says little, and that little is guarded, and consistent with Mr. Brodie's practical caution. He believes that the large doses in which iodine is commonly exhibited in this country are actually dangerous. He thinks that the medicine cannot be taken constantly, and that it may properly be made to alternate with courses of steel, or that the two may be advantageously combined. It is, indeed, lamentable to perceive the infatuation which some practitioners labour under respecting iodine. For all cases which are scrofulous, or can by any ingenuity be thought to be so—for all indolent tumors—iodine, in large doses, is frequently prescribed as a matter of course, and without a suspicion that a surgeon, of any judgment, would hesitate to order it. Such is the force of habit on the mass, that the instant a remedy acquires reputation, genuine or false, it is universally and blindly distributed. Mr. Brodie remarks that, when the secretions are unhealthy, occasional mercurial alteratives are useful; but mercury, in larger doses, is invariably prejudicial. Mr. Brodie makes no reference to sarsaparilla. Could we trust our own experience, we should say it was more valuable, when properly exhibited, than even steel.

But local treatment, though subsidiary to constitutional management, is far from unimportant.

Mr. Brodie is disposed to doubt the efficacy of large local bleedings; but moderate local depletion, in the early stage of the disease, or when accidental inflammation is set up, is rational, consistent with analogy, and supported by experience. Counter-irritants are very rarely useful, except in some cases of disease in the hip-joint, in which the patient suffers, in an unusual degree, from pain and muscular spasms in the limb.

But perfect quietude of the limb is always useful—always safe. There are no particular directions necessary in the management of the abscesses. Mr. Brodie seems to prefer their being allowed to burst. Yet sometimes great relief is experienced from opening them by means of the lancet. We have a case of scrofulous disease of the ankle-joint now under our care, in which this was certainly the case. When the disposition to suppuration appears to have ceased, pressure, cautiously applied by strips of soap plaster, gives support to the limb, and promotes the curative process of anchylosis.

"If a portion of the bone has lost its living principle, and has exfoliated into the cavity of the joint, the chance of ultimate recovery is very much diminished. For the most part, the dead bone is so entangled in the living parts, that it is incapable of separation by a natural process; and every attempt to remove it by artificial means will occasion a fresh attack of inflammation and abscess. It is to be observed, however, that bone which is found exposed at the bottom of a sinus is not necessarily doomed to exfoliate. It may be simply ulcerated, and may possibly granulate, and recover; and the surgeon, therefore, is not warranted in giving a prognosis which is decidedly unfavourable, merely because he discovers a piece of exposed bone, when he makes an examination with a probe." 207.

The question of amputation next presents itself—one attended with more difficulty than the hasty or the inexperienced surgeon might be tempted to imagine. On this point, the opinions of our author are highly deserving of attention.

When the joint is destroyed and the health is failing, no doubt can be entertained of the propriety of the operation. But the health may have suffered comparatively little, and yet the condition of the joint may be such as to render ultimate recovery very doubtful, and that, when it occurs, no better than anchylosis, after a considerable lapse of time. Is amputation preferable to such imperfect preservation of the limb? To determine this, it is necessary to consider two orders of facts of no trivial consequence. Those facts are in some measure opposed—they require sound judgment to balance, to compare, and to decide upon them. For, first, cases shew that, after the removal of a scrofulous joint, disease of internal organs supervenes; and, secondly, cases also seem to prove, that a scrofulous joint allowed to remain too long, does appear, of itself, to give rise to visceral disease. Let us hear

Mr. Brodie on this subject. Its interest must excuse the length of the quotation.

"A girl," says he, "was admitted into St. George's Hospital, who laboured under this disease in the bones and joints of the tarsus. Her foot was amputated by Mr. Griffiths. In about three weeks the stump was perfectly healed; but now she was seized with symptoms which indicated an affection of the mesenteric glands, which had not shewn itself previously, and she died. On dissection, numerous glands of the mesentery were found enlarged, and containing a cheesy matter. Another girl, whose arm I amputated on account of a scrofulous disease of the elbow, became affected in the same manner immediately after the stump was healed. She also died, and similar appearances presented themselves on dissection. A man, whose leg was amputated on account of a scrofulous disease of the tarsus, in a short time after the operation began to experience symptoms which indicated the incipient state of some pulmonic complaint: and soon afterwards the other foot became affected in the same manner as the first. These are a few of many cases which might be adduced, as leading to this conclusion,—that the occurrence of this scrofulous disease, in a particular joint, may be the means of preventing the scrofulous disposition from showing itself in some other organ; and that if the affected joint be removed by an operation, there is more danger of disease breaking out elsewhere, than there would have been if the operation had not be resorted to.

But we may refer to another order of facts, as shewing, that there are occasions in which the amputation of a scrofulous joint, instead of rendering other organs more liable to the same disease, may actually produce the opposite effect of preserving them from it. It is to be observed that such a disease of a joint is never more than the remote cause of death, and that, where the result is fatal it invariably happens in the following manner. The patient is exhausted by a hectic fever, and, in this state of debility, disease takes place in the mesentery or lungs, or not unfrequently in both these parts at the same time, and it is this visceral affection which immediately precedes dissolution. It is evident, then, that in many cases there is a period of time at which the amputation of the limb may be the means of preventing the establishment of a secondary disease. Nor is this all. Visceral disease, which was previously in a state of inactivity, may assume a new form, and begin to make a rapid progress, under the influence of the disease of the joint; and amputation, under these circumstances, may be the means of preserving the patient, if not altogether, at least for a considerable time, perhaps for several years. A young woman was admitted into the hospital labouring under a scrofulous affection of the ankle. It was of long standing, and there were several abscesses communicating with extensive surfaces of carious bone. It was evident that there was no chance of cure for the disease in the joint. Nevertheless I did not think it right to propose to the patient that she should submit to the loss of the limb, as she had a troublesome cough, with a purulent expectoration, and other marks of pulmonary disease. She, however, earnestly implored that the ankle might be removed, and at her request, and certainly against my own judgment, I performed the operation. The stump healed readily. The pulmonary symptoms almost immediately subsided. She lived for four or five years in tolerable health, but at the end of that period, (as I have been informed,) there were again manifest indications of disease within the chest, of which she ultimately died.

It is evident, from these statements, that the question concerning amputation is, in many instances, one of a complicated nature, requiring the exercise of no small degree of judgment and discrimination on the part of the surgeon, and not to be determined, except after a minute investigation of the whole case, with respect to the disease in the joint itself, and also in whatever relates to the state of the general health at the time, and that of the constitution previously." 212.

The only point remaining for our attention is anchylosis.

If, indeed, the disease has been arrested early this may not occur, a membranous substance, possibly even true cartilage, supplying the place of that which has been lost. In other instances adhesions are formed between the articulating surfaces. Usually their small extent is advantageous to the patient; but this is not always the case. For example, in the joint of the knee it is not uncommon to find the patella completely united to the condyles of the femur, while the head of the tibia admits of a considerable degree of flexion and extension. This partial degree of mobility is productive of no small degree of inconvenience, and the patient would, in fact, be in a much better state if the anchylosis were complete in every part, as, in consequence of the fixed state of the patella, he has no power to act on the leg by means of the extensor muscles. The joint is indeed moveable, but its motions are not under the control of the will.

When recovery takes place after the formation of an abscess communicating with the joint the adhesions are general, the anchylosis complete. Bony anchylosis is rare, and at all events is not established till after the lapse of many years. It is never prudent to resort to any mechanical means for the purpose of preventing anchylosis, but we may venture, when the circumstances of the case require it, to adopt measures for gradually improving the position of the limb. For instance: when the knee has been affected, if left to itself, it often happens that the leg becomes fixed at a right, or even an acute angle with the thigh; and a light apparatus may be applied to the limb, with a screw at the posterior part, by the agency of which the leg may be very slowly and cautiously extended. In like manner, if the elbow be in danger of being anchylosed in the straight position, it may be very gradually brought into a state of flexion.

Mr. Brodie concludes an account of some cases of this disease, with some remarks which merit the attention of the pathologist. In referring to the morbid changes he observes, that the softening of the cancellous structure is only one of the alterations effected by scrofula, and we must not hastily conclude that this is always the original malady where we meet with bones thus deprived of their earthy matter. It is certain that a preternatural softness may occur after inflammation and caries of a bone which was previously healthy. It is only by considering the series of changes and concomitant circumstances that we can properly pronounce on the nature of the alteration. In noticing the former chapters of this work, we concluded each with one or two cases exhibiting the symptoms and the treatment. We shall take the liberty of adopting a similar method here, although we are aware that our analysis has extended to a considerable length.

*Case.* A scrofulous looking boy, æt. 6, was admitted into St. George's Hospital, in February, 1814, on account of an affection of the left knee. It was an inch and a half in circumference larger than the other. The swelling was puffy and elastic; without fluctuation: having nearly the form of the articulating extremities of the bones; but filling up the space on each side of the ligament of the patella. The joint admitted of considerable motion, but not of complete flexion and extension. He complained of pain, which was worst at night; but never very severe. It was somewhat aggravated by pressure.

His parents attributed the complaint to some trifling hurt, which he had met with a year ago; soon after which, a slight degree of pain and tumefaction was first observed, which had continued ever since, and had increased, particularly within the last month.

The treatment adopted in the hospital consisted of leeches and cupping in the first instance—the internal use of the vinum ferri with the tinctura ferri muriatis—and in March, the application of soap strapping to the joint, with the view of restraining its motions without interfering with the patient's exercise. At the latter end of April there was neither pain nor stiffness of the joint, and scarcely any swelling. The patient then quitted the hospital.

*Case.* A scrofulous-looking boy, æt. 9, complained in January, 1817, of an aching in his left elbow, which in two or three months was observed to be swollen. In May, an abscess formed and burst, on the inside of the elbow, and in January, 1818, another abscess began to shew itself upon the outside. He was now received into the hospital. The joint admitted of very limited motion. Whenever it was moved, or when the articulating surfaces were pressed against each other, he complained of some, but not of severe pain. He kept the fore-arm in the half-bent position, and walked about, supporting the hand in a sling, with very little inconvenience.

In the beginning of February, he was directed to take six grains of carbonate of iron three times in the day; and a purge of calomel and rhubarb was administered occasionally. The abscess was opened, and a poultice was applied. In May there was another abscess, and in June another, one of which was opened, and the other broke. In July there was little or no swelling—he was free from pain—and the abscesses, though open, discharged a very small quantity of matter. The poultices and fomentations were now discontinued, and some simple dressings and a bandage were applied. In September, the joint was not larger than the other; it admitted of much more motion than formerly; there was no pain; there was still one sinus, which was not completely closed, and which discharged a minute and almost imperceptible quantity of matter; all the other abscesses were completely healed.

The next case illustrates more perfectly the practice which Mr. Brodie has adopted of late years.

*Case.* Master H. K. then two years of age, was taken to Mr. Brodie in December, 1831.

The right knee was enlarged. The leg was half bent on the thigh, and the joint admitted of motion to only a limited extent. The swelling manifestly arose, not from fluid in the cavity of the synovial membrane, but from an effusion of lymph and serum in the cellular membrane external to it. The projecting condyles of the femur presented the usual rounded appearance which is observable in cases of the scrofulous disease of this articulation. The child complained very little, or not at all, of pain. There were no marks of derangement of the general health. The complaint had begun about the end of the previous October.

A pasteboard splint was applied on each side of the joint; the *vinum ferri* was prescribed to be taken twice daily for three weeks or a month, then omitted for a week or ten days, and then to be given for a month again, and so on. It was also directed, that some calomel should be administered once in three weeks, with an occasional dose of rhubarb and sal polychrest in the intervals; that he should be taken back into the country; that he should be drawn out of doors in an open carriage, so as to be exposed to the fresh air for some hours, daily, in fine weather; and lastly, that he should be prevented, as much as possible, from using the limb.

In May, 1832, a swelling appeared on the inside of the thigh above the knee, which, in September, had all the appearance of an abscess. In May, 1833, this swelling was much reduced, and that of the knee was diminished also. In June, the former had disappeared. The diseased knee was no larger than the other, but it was stiff, and the leg was bent at a right angle with the thigh.

It was now directed that the splints should be left off, and that an instrument should be applied at the back part of the limb, attached to the thigh and leg, so as to give much support to the joint, at the same time that it was furnished with a screw, by means of which the leg might be very cautiously and gradually extended. No change was made in the treatment in other respects.

The machine completely answered the purpose for which it was intended. In a fortnight after it was first applied, the little boy was able to walk across the room without difficulty, and altogether it was so convenient that he was allowed to wear it during the night, by his own express desire.

In January, 1834, the knee was reduced to nearly its natural size. The leg was bent only in a very slight degree, and the patella moved readily upon the femur. The boy could walk pretty well with the assistance of the instrument. During the whole of the previous period, the patient had been kept in the country, being merely brought to town occasionally for the benefit of Mr. Brodie's advice. He was now directed to return to the country and to follow precisely the same plan of treatment.

Here we must close this notice. We have arrived at the 230th page of the volume, and have presented to our readers a close delineation of the portion we have traversed. Upwards of a hundred pages remain for analysis—and several subjects of no mean importance are left for consideration. Those subjects are caries of the spine—tumors and loose cartilages in the cavities of the joints—malignant and some other diseases of the latter—inflammation of the bursæ mucosæ.

On looking back at what has been noticed, we may spare ourselves the pleasure of addressing praise to the author. We will merely observe, that we have presented to our readers, perhaps in as small a compass as possible, a mass of as valuable information as any contained in a medical or surgical work. We return to the analysis of the volume before us in the next number of this Journal.

